# Mechanisms and Functions of the RNA Polymerase II General Transcription Machinery during the Transcription Cycle

**DOI:** 10.3390/biom14020176

**Published:** 2024-02-01

**Authors:** Stephen R. Archuleta, James A. Goodrich, Jennifer F. Kugel

**Affiliations:** Department of Biochemistry, University of Colorado Boulder, 596 UCB, Boulder, CO 80309, USA; stephen.r.archuleta@colorado.edu

**Keywords:** transcription, RNA polymerase II (Pol II), general transcription factors (GTFs), promoter, preinitiation complex

## Abstract

Central to the development and survival of all organisms is the regulation of gene expression, which begins with the process of transcription catalyzed by RNA polymerases. During transcription of protein-coding genes, the general transcription factors (GTFs) work alongside RNA polymerase II (Pol II) to assemble the preinitiation complex at the transcription start site, open the promoter DNA, initiate synthesis of the nascent messenger RNA, transition to productive elongation, and ultimately terminate transcription. Through these different stages of transcription, Pol II is dynamically phosphorylated at the C-terminal tail of its largest subunit, serving as a control mechanism for Pol II elongation and a signaling/binding platform for co-transcriptional factors. The large number of core protein factors participating in the fundamental steps of transcription add dense layers of regulation that contribute to the complexity of temporal and spatial control of gene expression within any given cell type. The Pol II transcription system is highly conserved across different levels of eukaryotes; however, most of the information here will focus on the human Pol II system. This review walks through various stages of transcription, from preinitiation complex assembly to termination, highlighting the functions and mechanisms of the core machinery that participates in each stage.

## 1. Introduction

Controlling gene expression is essential to normal growth, development, and sustained life. In metazoans, this requires regulating the spatial, temporal, and developmental expression of genes in a wide diversity of cell types. Mis-regulation of gene expression contributes to most disease states. The main control point for regulating gene expression is at the level of transcription. In eukaryotic cells, RNA polymerase II (Pol II) transcribes protein-coding genes into messenger RNA (mRNA) transcripts. Pol II also synthesizes long non-coding RNA (lncRNA) and most small nuclear RNA (snRNA) and microRNA (miRNA). Pol II transcription is vital for cell proliferation, proper expression of metabolic enzymes, signaling, cell fate, differentiation, gene expression, and nearly every cellular process. Although the RNA polymerase II transcription system is highly conserved across eukaryotes, this review is primarily focused on the human system, with some references to data from Drosophila and yeast systems.

The Pol II core enzyme can itself synthesize RNA using a template DNA, but promoter-specific transcription initiation requires the canonical general transcription factors (GTFs): TFIIA, TFIIB, TFIID, TFIIE, TFIIF, and TFIIH (Table 1). In addition, the large multi-subunit complex Mediator is essential for proper transcription in cells [1,2]. Chromatin remodeling/modifying complexes and additional co-regulatory factors function together with promoter-specific transcriptional activators and repressors to set the proper level and timing of transcription from individual genes in specific cell types. This review serves as an overview of the general factors involved in the Pol II transcription reaction. This includes factors central to the steps of preinitiation complex assembly, promoter melting, initiation, early transcription, elongation, and termination. The roles of Mediator [1,2], chromatin regulators [3,4], transcriptional activators, repressors and enhancers [5,6], and co-regulatory complexes [7,8] in controlling transcription have been reviewed elsewhere.

## 2. RNA Polymerase II

Pol II is a large ~500 kDa complex made up of 12 protein subunits, named Rpb1-12. Studies have shown that 10 of the 12 subunits form the catalytic core of the Pol II complex and are either identical (Rbp5, 6, 8, 10, 12) or highly similar (Rpb1-3, 9, 11) to subunits found in RNA polymerase I and RNA polymerase III, which transcribe primarily tRNAs and rRNAs, respectively [9,10]. Recent ChIP-seq (chromatin immunoprecipitation followed by high-throughput sequencing) and mass spectrometry studies have shown that different sets of Rpb subunits differentially regulate select subsets of human genes, demonstrating the dense layers of regulation within the Pol II complex itself [11]. Crystal structures of yeast Pol II, and more recently cryo-EM structures of human and yeast Pol II, have revealed that the Pol II complex can be divided into the core, shelf, jaw lobe, and clamp structural domains that interact with each other and undergo conformational changes during the stages of transcription (these structures have been extensively reviewed in the literature [12,13,14,15]). The core domain contains Rpb3 and Rpb10-12 as well as the positively charged active center cleft, formed by Rpb1 and Rpb2 [16,17]. The active site of Pol II is buried deep at the base of the active center cleft, thus requiring translocation of the template DNA strand to the active site after entering the cleft. The shelf and jaw lobe elements have little observed movement but can rotate parallel to the active center cleft [17]. The clamp domain is connected to the active site cleft in the core domain through an array of flexible switches, and it swings nearly 30 Å upon opening or closing the cleft [9,17]. While not considered part of the catalytic core of Pol II, binding of the Rpb4 and Rpb7 subunits has been shown to be vital for maintaining the closed conformation of the Pol II clamp over the DNA during initiation [18,19]. It is hypothesized that the closing of this clamp domain over the cleft coupled with DNA distortion may facilitate promoter melting [20].

At 250 kDa, Rpb1 is the largest of all Pol II subunits and the principle catalytic subunit of the Pol II complex [21]. Beyond its catalytic role, Rbp1 plays a regulatory role in the transcription cycle that is mediated by the unstructured C-terminal domain (CTD) on the Rpb1 subunit. The CTD consists of a long tail comprising heptapeptide repeats of the consensus sequence YSPTSPS, with minor variability at the Ser7 position in repeats near the C-terminus [22,23]. Mammalian Pol II contains 52 repeats, with the number of repeats varying among different organisms in a manner that loosely correlates with genomic complexity [24]. The YSPTSPS consensus sequence is well conserved across eukaryotes, emphasizing the functional importance of each residue [24]. The CTD tail is not necessary for basal (i.e., unregulated) Pol II transcription in vitro [25,26]; however, it is required for accurate Pol II transcription and proper termination in cells [27,28,29]. The CTD is thought to function as a binding platform for association of numerous other protein complexes that help regulate co-transcriptional processes or steps in transcription, including RNA splicing and transcription termination [30,31,32].

**Figure 1:**
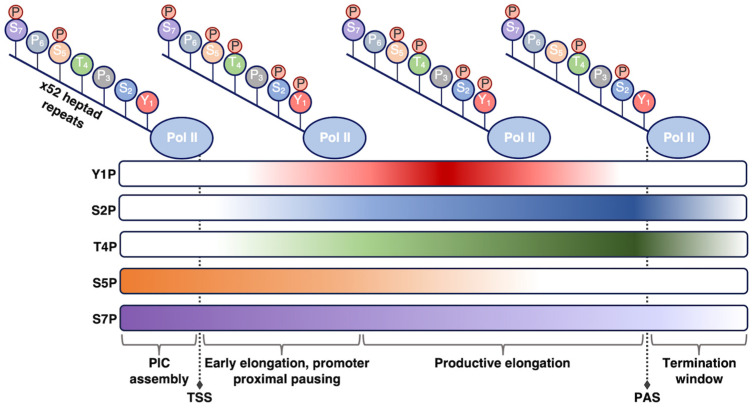
Phosphorylation state of the Pol II CTD is regulated during transcription. As Pol II transcribes through a gene and progresses through the stages of transcription (shown from left to right), different phosphorylation marks are added or removed to promote unique functions. The phosphorylation patterns shown here pertain to human Pol II; other organisms may exhibit slight differences in these patterns. TSS, transcription start site; PAS, polyadenylation site.

The residues within the heptapeptide repeat are substrates for many post-translational modifications, with phosphorylation being the most well characterized. The Tyr, Ser, and Thr residues can be reversibly phosphorylated/dephosphorylated, allowing for regulation of Pol II activity through the transcription reaction and of Pol II CTD affinity for various regulatory factors [22,23,30] (Figure 1). For example, the level of specific phosphorylation marks varies across different stages of transcription, depending on the purpose of the modification. The use of phospho-specific antibodies coupled to ChIP-seq, in addition to in vitro work, have enhanced understanding of how CTD phosphorylation patterns change throughout the transcription cycle. Pol II is recruited to preinitiation complexes on promoter DNA in a hypo-phosphorylated form. Phosphorylation of Ser5 by the CDK7 kinase subunit of TFIIH (which is one of the general transcription factors discussed below) facilitates initiation of transcription. The Ser5 mark is removed as Pol II moves throughout the gene body. As a counterpoint, Ser2 phosphorylation predominantly accumulates after initiation to help recruit elongation and RNA processing factors and peaks at the 3’ ends of genes where it is thought to facilitate termination [33,34,35,36]. Beyond Ser5 and Ser2, other sites of phosphorylation on the Pol II CTD include Tyr1, Thr4, and Ser7, which have not been studied in the same detail as the other CTD residues. Research has shown that the significance of these three residues can vary across species, with metazoan and yeast systems sometimes exhibiting different behaviors [37,38]. Exploring the function of these other important Pol II CTD residues provides numerous areas for future study.

## 3. Preinitiation Complex (PIC) Formation and the GTFs

### 3.1. PIC Assembly Mechanisms

**Figure 2:**
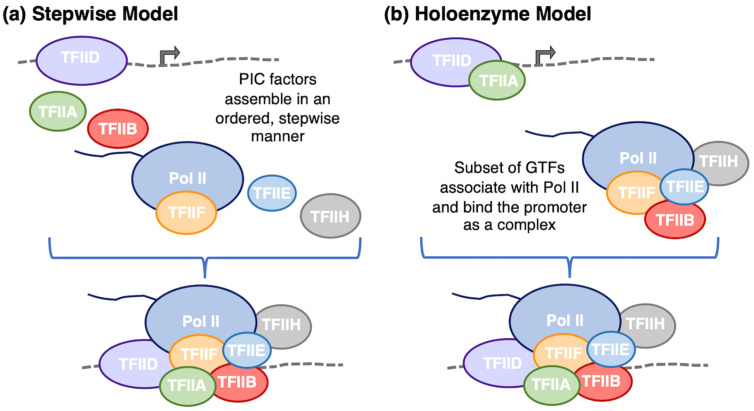
Two mechanisms for PIC formation, which are not mutually exclusive. (**a**) In the stepwise model, Pol II and the GTFs assemble in a particular order facilitated by one factor recruiting the subsequent factor via protein–protein interactions. (**b**) In the holoenzyme model, minimally TFIID and TFIIA assemble at the promoter while Pol II and remaining GTFs form a subcomplex that binds to the promoter, completing PIC assembly.

Eukaryotic Pol II does not have sequence-specific DNA binding capacity. Therefore, it relies on an array of GTFs to properly position Pol II at the core promoter region of genes, which contains the transcriptional start site (TSS). The GTFs assemble along with Pol II into a PIC in a tightly regulated process. Decades of research using in vitro biochemical assays, cellular systems, and structural approaches have provided a wealth of fundamental insight into how PIC formation occurs; however, the precise mechanisms and pathways are not yet fully defined. In general, there are two distinct, but not mutually exclusive, models for how PIC assembly occurs (Figure 2) [39,40]. In the stepwise assembly model, the GTFs assemble on the promoter DNA in a sequential order: TFIID/TFIIA, TFIIB, Pol II/TFIIF, TFIIE, then TFIIH. This model was initially developed from in vitro biochemical studies that recombined the purified GTFs in different orders, monitoring complex assembly and transcriptional activity [41,42]. In the holoenzyme model of assembly, Pol II and many of the GTFs pre-assemble off of the promoter DNA and bind as a unit to the core promoter. Evidence supporting the holoenzyme model of assembly arose from experiments showing that Pol II co-purifies in a complex with various subsets of GTFs in the absence of DNA [43]. While the subset of GTFs isolated as part of the Pol II holoenzyme varies within the literature, one study found that TFIID and TFIIA were absent from the holoenzyme [44], suggesting that TFIID and TFIIA first bind the promoter, then recruit the Pol II/GTFs holoenzyme to form a complete PIC. It is likely that both models of assembly occur in cells, and assembly is regulated to enable specific transcriptional responses at different promoters in response to unique cellular stimuli [40].

Recent advances in our understanding of PICs come from the advent of new technologies such as cryo-EM and single-molecule imaging. A profusion of cryo-EM studies in recent years has provided detailed pictures of the architecture of PICs and other transcription complexes, many of which are referred to in other sections of this review. Importantly, advances in this technique allow multiple conformations of complexes to be resolved to provide insight into protein flexibility. Due to the large size and number of individual protein subunits that comprise PICs, capturing this flexibility informs how assembly and early steps in transcription are facilitated by specific protein–protein interactions. This body of structural work has been extensively reviewed elsewhere [12,13,14,15,45,46].

Recent advances in single-molecule imaging in vitro and single-particle tracking in live cells have also advanced our understanding of PIC formation and general transcription machinery. These studies visualize fluorescently labeled protein factors in real-time with millisecond resolution to provide a dynamic view of how factors interact with each other and the genome as well as heterogeneity in their behavior. Below we describe examples of how these findings have advanced our understanding of transcriptional control, focusing on general transcription machinery. Although outside the scope of this review, single-molecule imaging studies are also breaking new ground in understanding mechanisms of transcriptional regulation and how transcriptional activators function (for reviews, see [47,48,49,50]). For example, real-time imaging has shown that, in general, transcription activator binding is very dynamic with brief residence times on chromatin and different kinetic populations that likely reflect functional and non-functional interactions (for examples, see [51,52,53,54,55]). The imaging approaches and findings surrounding transcriptional activators will be informative for ongoing single-molecule studies of the general transcription machinery.

The ability of single-molecule imaging to resolve heterogeneity and rapid dynamics has provided new insight into behaviors of the GTFs and Pol II. For example, one study using reconstituted human PICs found that TFIIB binding in the PIC is highly dynamic and only becomes stably bound after Pol II/TFIIF is recruited to the PIC [56]. Another in vitro work revealed that the release of TFIIB after synthesis of a 7- or 9-nucleotide RNA is tightly coupled to maintaining the activity of complexes [57]. Work using yeast nuclear extracts with fluorescently labeled factors showed that Pol II can assemble with TFIIE and TFIIF on an upstream activation sequence before being transferred to the core promoter with the other GTFs [58,59]. Single-molecule tracking of PIC components in live yeast cells found that the GTFs will sub-diffuse through a small space in the nucleus, confined by the large complexes of Mediator and TFIID [59,60]. Imaging Pol II itself in human cells has informed our understanding of how the polymerase tracks through the stages of transcription [61,62]. Measuring the turnover rate of different Pol II populations near promoters showed Pol II molecules freely diffusing, bound to chromatin, paused at the promoter, or productively elongating [63].

Imaging Pol II and associated factors in human cells has also detailed spatiotemporal regulation of transcription through transcriptional bursting and Pol II clustering. Transcription is discontinuous over time, giving rise to fluctuations in activity known as bursting, which is supported by clusters of Pol II that form highly dynamic foci [64,65]. Studies show the Mediator complex can load several Pol II enzymes onto the promoter region to form a convoy of polymerases that initiate and enter productive elongation [66]. Moreover, data show that dynamic clusters of Pol II, Mediator, and other cofactors can form condensates [67,68]. These spatiotemporal phenomena appear to form the basis of a rapid response transcription system activated by cellular stimuli. Indeed, recent work shows that transcriptional condensates can form at enhancers to amplify the magnitude and frequency of transcriptional bursting when in proximity to a gene [69]. These important mechanisms of Pol II function and regulation are reviewed in detail elsewhere [64,70,71].

### 3.2. TFIID and the Core Promoter

PICs are nucleated by the general transcription factor IID (TFIID) bound to the promoter DNA. A recent comprehensive study of active promoters in human cells concluded all promoters are organized around the purpose of serving as a TFIID binding site [72]. TFIID is a large 14-subunit complex composed of the 37 kDa TATA-binding proteins (TBPs) and 13 TBP-associated factors (TAFs) ranging in size from 15–250 kDa [39]. The TBP subunit specifically binds to the minor groove of the TATA box, a core promoter DNA element located upstream of the TSS with an AT-rich consensus sequence that has been defined in vitro and more recently in human cells [73,74]. Upon binding, TBP induces a sharp ~90° bend in the DNA via phenylalanine residues intercalating between the DNA base pairs near each end of the TATA box [75,76]. This sharp bend helps position the DNA with respect to Pol II, which may explain why TBP or a TBP homolog is required for basal transcription at genes transcribed by all three RNA polymerases [77]. Interestingly, TBP is able to bend DNA when bound to promoters with or without a TATA box, highlighting the importance of this behavior for transcriptional activity [78]. Structural data suggest that DNA bending repositions factors at the promoter in a conformation that enables contacts that are not possible on a linear DNA conformation, and it helps to modulate the auto-repressive nature of some of the TAF subunits on TBP [13]. However, the large majority of human genes lack a TATA box near their transcriptional start sites [79,80,81,82], including many housekeeping genes and genes encoding growth factors, transcription factors, and oncoproteins [83]. A recent study utilizing rapid protein depletion followed by PRO-seq found that TBP is dispensable for driving transcriptional activity at promoters that do not contain a TBP-binding motif, suggesting the importance of other factors for promoter recognition [74].

Without a TATA box, promoter recognition must occur via interactions mediated by the TAF subunits of TFIID. The TAFs recognize other promoter elements, most notably the initiator (Inr) element [84]. The Inr surrounds the TSS and is sufficient for initiation in the absence of other promoter elements [82,85]. Inr is thought to be present at most human promoters outside of ribosomal protein genes [72]. Sequences downstream of the TSS are also important for promoter recognition by TFIID. Several downstream promoter elements have been characterized in Drosophila systems, but their presence in human systems is poorly understood. However, studies that incorporated computational modeling, sequencing techniques, and functional studies have found evidence for active downstream promoter motifs in human systems [86,87]. Moreover, evidence of multiple TAFs binding downstream of the TSS has been found using crosslinking [88], cryo-EM [89], ChIP-exo experiments, and in vitro transcription assays followed by quantitative mass spectrometry [90]. Structural studies suggest that the TAF subunits may change conformations, or even dissociate, after depositing TBP at the promoter due to predicted steric clashes with GTFs and Pol II that subsequently assemble into the PIC [91]. TFIID, and particularly TAF1, is also shown to be involved in promoter proximal pausing and positioning of the +1 nucleosome downstream of the TSS [72,92]. The binding and proper alignment of TFIID on the core promoter is critically important for PIC nucleation and regulation of early transcription [93].

Our understanding of core promoter complexity has evolved in recent years due in large part to information gained from diverse applications of high-throughput sequencing approaches. Active core promoters exhibit different initiation patterns defined in part by TSS selection. Genes that initiate transcription over a broad region from multiple transcription start sites exhibit “dispersed/broad” initiation, whereas genes following the canonical structure of a single predominant TSS have “focused/sharp” initiation [84,94]. Focused genes are typically those that are tightly regulated or cell type-specific, whereas dispersed genes are often broadly expressed across many cell types, such as housekeeping genes. It is believed that dispersed promoters in human cells function by allowing for the assembly of multiple PICs with defined TSSs that together define a transcriptional start region, in contrast to yeast where a single PIC may drive initiation from multiple TSSs [72]. These two broad promoter classes have many important differences, including arrangement/presence of core promoter motifs, placement of the +1 nucleosome downstream of the TSS, behavior of promoter proximal pausing, and associated coactivators, among others [95,96]. Another added layer of complexity present at core promoter regions is bidirectional or divergent transcription, in which two separate Pol II complexes initiate transcription in opposite directions [97,98]. At protein coding genes, this process produces the mRNA plus an unstable upstream noncoding RNA (ncRNA) in the antisense orientation [99,100,101]. Enhancers, which are regions of DNA that contain binding sites for sequence-specific transcriptional activators/repressors, are also sites of bidirectional transcription. Signals of bidirectional transcription at enhancers indicate their active influence in gene expression [102,103]. This process generates enhancer RNAs (eRNAs) that can influence gene expression [97]. The regulatory network between promoters, Pol II, TFIID, and other general factors continues to provide a rich field for new discoveries.

### 3.3. TFIIA

The main role of TFIIA during transcription is the stabilization of the TFIID–DNA interaction. TFIIA is a heterotrimer consisting of α, β, and γ subunits with masses of 35, 19, and 12 kDa, respectively, and binds just upstream of the TATA box. TFIIA makes direct contacts with TFIID, TBP, TFIIE, and TFIIF within the PIC [89,104]. TFIIA also binds to several activators and repressors, and it can enhance the effects of co-activators [105]. TFIIA binding to activators and TFIID has been proposed to hasten TFIID recognition of the promoter DNA, a typically rate-limiting step [106]. TFIIA has also been shown to increase TBP affinity for the TATA box [42,107], especially in conditions where TBP binding to promoter DNA is suboptimal [108]. Free subunits of TBP or TFIID can form homodimers in solution, likely as a way to regulate the rate of promoter recognition and PIC assembly; TFIIA can facilitate dissociation of TBP and/or TFIID homodimers, thereby accelerating promoter recognition [109]. TFIIA can be cleaved by Taspase1, and both cleaved and uncleaved forms can exist in cells. Cleavage is not necessary for activity, but it does impact turnover rate of TFIIA in cells, which may be a source of regulation [105]. The cleaved and uncleaved forms have different affinities for TBP and can form unique sub-complexes, which also have different affinities for promoter DNA and/or chromatin [110]. Therefore, the cellular concentration of TFIIA, the ratio of cleaved and uncleaved TFIIA, and its ability to bind to TBP/TFIID play critical roles in the regulation of PIC nucleation. Mutational and depletion studies targeting the TBP–TFIIA binding interface in yeast showed a decrease in transcriptional activity at various promoters, suggesting the impact of TFIIA on transcriptional output is promoter-specific [111,112]. Together, the literature surrounding TFIIA shows it supports transcription through diverse mechanisms that facilitate PIC assembly, recruitment of GTFs, and interactions with co-transcriptional activators and repressors.

### 3.4. TFIIB

TFIIB is a single-subunit (33 kDa) protein. In the ordered mechanism of PIC assembly, TFIIB is recruited to stabilize the DNA–TBP–TFIIA complex, then recruits Pol II/TFIIF to the PIC [39,40]. In addition, TFIIB helps specify the TSS and orient Pol II binding to ensure proper PIC directionality [113]. Within the core promoter, TFIIB recognizes the upstream (−38 to −32) and downstream (−23 to −17) TFIIB recognition elements (uBRE and dBRE, respectively) that surround the TATA box [114,115]. The BREs are often found in TATA-less promoters, allowing TFIIB to enhance TFIID binding in the absence of the TATA box [79]. While TFIIB can bind to the uBRE in the absence of TBP, recognition of the dBRE requires prior binding of TBP [114,115].

TFIIB has multiple important functional domains, including the B core domain, N-terminal B ribbon region, and B reader domain. The B core domain contains two cyclin repeats that recognize the BREs and properly position TFIIB on the promoter. The two cyclin repeats of TFIIB do not have the same affinity for the TBP–DNA complex, leading to a strong preference for a single orientation of TFIIB binding and thus ensuring that initiation cannot erroneously occur upstream of the TATA element [116,117]. A yeast Pol II-TFIIB co-crystal structure revealed that TFIIB positions the promoter DNA near the Pol II cleft to allow for active site access and stabilization by downstream GTFs [19]. The B ribbon functions as a molecular switch governing conformational changes taking place within the B core cyclin repeats upon DNA binding [118,119]. The B reader domain contains multiple motifs, including the B reader helix, loop, and strand regions that directly interact with Pol II. A yeast co-crystal structure showed the B reader loop extends into the mRNA exit channel of Pol II, possibly guiding the nascent RNA away from the template [120]. Obstruction of the exit channel suggests that TFIIB must either change conformation or release from the complex during early elongation. In vitro single-molecule studies demonstrated that synthesis of 7- and 9-nucleotide RNA transcripts triggers TFIIB release, as predicted by previous structural data [57]. Multiple structural changes throughout initiation and early transcription, combined with direct contacts with Pol II, TBP, and TFIIF, emphasize TFIIB as a critical participant in regulating the transcription reaction.

### 3.5. TFIIF

TFIIF was first identified as RNA Pol II-associated proteins, hence the RAP designation for its two RAP30 and RAP74 subunits [121]. TFIIF is thought to associate with Pol II away from the promoter DNA [122,123]. This interaction strongly inhibits non-specific DNA binding and initiation by Pol II, analogous to the bacterial σ factor [124]. Structural studies show a charged helix domain of RAP74 binding Rpb2 in the Pol II lobe, Rpb9 in the Pol II jaw, and Rpb1 [78,91,104]. Photo-crosslinking studies localized RAP30 just downstream of TBP (−19) and RAP74 just upstream of the TSS (−15 to −5) [125]. Data obtained using other structural methods agree with this positioning of RAP30 and show its winged-helix domain cooperating with TFIIB to stabilize the promoter DNA between −23 and −13 [78,91,126]. Both TFIIF subunits contain winged-helix domains with strong DNA-binding activity, which enhances Pol II/TFIIF affinity for the promoter and increases the overall stability of the PIC [127,128]. TFIIF also induces important structural changes in the PIC and topological changes in the DNA upon recruitment, including wrapping the DNA around Pol II [91,128].

Biochemical studies support a unique role for TFIIF as an early Pol II elongation factor by enhancing the transition to productive elongation and facilitating early RNA synthesis via suppressing abortive transcription [129,130]. Data suggest TFIIF increases the rate at which the first few phosphodiester bonds are formed, thus ensuring that nascent transcripts become long enough to resist abortive transcription [129]. TFIIF uses its position near the Pol II cleft to help maintain proper alignment of the 3′ end of the mRNA transcript in the Pol II active site, which helps prevent Pol II backtracking [131]. TFIIF is believed to remain associated with the polymerase through early transcription, as shown by results of biochemical studies [123,132,133], ChIP-qPCR [134], and ChIP-exo [132]. However, in cells this association is likely dynamic. Comparing structures of Pol II bound to various elongation factors reveals that many of these factors share overlapping binding interfaces on Pol II [135,136]. This mutual exclusivity due to shared binding sites allows for further temporal regulation of the early stages of transcript synthesis. Through its extensive network of protein–protein and protein–DNA contacts, TFIIF confers important stability to the PIC and regulates Pol II activity across early stages of transcription.

### 3.6. TFIIE and TFIIH

TFIIE consists of an αβ heterodimer with subunit masses of 56 and 34 kDa, respectively. TFIIE serves two main roles in PIC assembly: recruitment of TFIIH to the PIC and stimulation of multiple enzymatic activities of TFIIH [137,138,139]. TFIIEα binds strongly to TFIIH through its C-terminal domain, while the N-terminal domain is required for stimulation of TFIIH activities [137,140,141]. X-ray crystallography studies have shown that heterodimerization of TFIIE involves a winged-helix domain of TFIIEα directly contacting a winged-helix domain of TFIIEβ, and two coiled-coil helices that are intertwined with TFIIEα [142]. TFIIEα also contacts TBP and TFIIF, and it contains a short region homologous to the bacterial σ factor [137]. Using its zinc finger motif, TFIIEα enhances TBP binding at multiple promoter constructs in vitro and contacts the Rpb7 subunit of the Pol II stalk [104,126,143,144]. Biochemical studies have demonstrated TFIIEβ binding to Pol II, TBP, TFIIB, and TFIIF [139]. More specifically, some data show the winged-helix motifs of TFIIEβ contacting the winged-helix domain of RAP30 [104]. In addition to protein–protein contacts, TFIIEβ also makes extensive protein–DNA contacts; TFIIEβ contains three winged-helix motifs used to bind double-stranded promoter DNA just upstream of the TSS (−14 to −2) [91,125,145]. Along with a winged-helix domain of RAP30 and a winged-helix domain of TFIIEα, there are a multitude of stabilizing interactions upstream of the transcription start site that trap the DNA against Pol II [91,126]. TFIIEβ also has a basic helix–loop sequence that interacts with single-stranded DNA [141,145], which likely stabilizes the melted promoter DNA during open complex formation [146].

TFIIE exhibits more dynamic binding behavior than the other GTFs, with rather low stability within PICs [58,147]. TFIIE binding is also severely impaired at promoters without TFIIF and Pol II [58]. This lower stability has been suggested in structural data, where TFIIE cannot be resolved in the absence of TFIIH, or TFIIE must be added in excess to allow visualization [91,126]. This suggests cooperative and/or simultaneous binding of TFIIE and TFIIH, and that TFIIH increases stability of TFIIE in the assembling PIC. Multiple studies have also suggested that TFIIE plays a vital role in facilitating the polymerase in clearing the core promoter and transitioning from initiation to elongation [148,149,150].

TFIIH is a 10-subunit complex composed of two domains: the 7-subunit core domain and the 3-subunit CAK domain [151,152]. The core domain contains the XPB and XPD subunits with translocase and ATPase activities, while the CDK7 subunit of the CAK domain is responsible for TFIIH kinase activity. XPD forms a structural anchor between the core and CAK domains and is not enzymatically involved in Pol II transcription; however, XPD is involved in the DNA damage response via nucleotide excision repair [152,153,154,155]. Cryo-EM structures of free TFIIH suggest that the XPD subunit is at least partially inhibited when the CAK domain is present in the TFIIH complex, possibly serving as a source of regulation for the multi-purpose roles of TFIIH in DNA repair and transcription [151,156]. Upon binding to the PIC at the promoter, TFIIH undergoes conformational shifts and transitions to an active form, where XPB and CDK7 are then used during initiation [151]. The translocase activity of XPB is important for promoter melting, which allows Pol II to access the template strand DNA. Structural data have shown that the XPB subunit must displace a portion of the TFIID complex in order to contact the promoter DNA, thus allowing DNA opening to occur [78,89]. The kinase activity of CDK7 is critical for phosphorylation of the Pol II CTD at Ser5 residues, allowing for Pol II to transition to its hyper-phosphorylated form, which promotes initiation followed by promoter escape [157]. Both TFIIH enzymatic activities are points of regulation in the transcription reaction and are critical for productive transcription, as described in the following sections.

## 4. Promoter Melting and Initiation

Once the PIC has been properly assembled on the promoter DNA, the enzymatic activities of TFIIH facilitate promoter melting and initiation. For Pol II to access the template DNA strand, the double-stranded promoter DNA must first be melted around the TSS utilizing the ATP-dependent translocase (XPB subunit) activity of TFIIH. XPB contacts the DNA downstream of the TSS (+10 to +20) and uses its 5′ to 3′ translocase activity to twist the non-template DNA strand, while pushing it back toward the Pol II cleft [14,78,104,126,158]. Since the DNA is bound by TBP, TFIIA, and TFIIB upstream of the TSS, translocating the DNA toward the Pol II cleft causes mechanical and torsional strain. This facilitates DNA unwinding around the transcription start site, forming an open DNA conformation/transcription bubble. GTFs bound to the promoter stabilize the open transcription bubble to prevent reannealing of the separated DNA strands [159]. TFIIE and TFIIF together form a surface of four winged-helix domains that stabilize the single-stranded DNA and prevent it from leaving the Pol II cleft [104,126]. The template DNA strand then moves into the base of the Pol II cleft where the active site resides [126,152]. Pol II utilizes free NTPs and metal ion catalysis to initiate transcription and synthesize the first phosphodiester bond of the mRNA [160,161]. The Pol II active site then translocates to the next position on the DNA template for the next round of catalysis (i.e., NTP binding, phosphodiester bond formation, and translocation) [162].

Also important for initiating complexes is phosphorylation of the Pol II CTD on Ser5 residues. TFIIH utilizes the CDK7 kinase of its CAK domain to phosphorylate the heptapeptide repeats at Ser5 [152]. Within a PIC, the CTD is thought to make extensive contacts with Mediator to help position Pol II, and phosphorylation by CDK7 disrupts these contacts, facilitating the transition to initiation [163]. In addition, multiple studies have shown that Ser5 phosphorylation recruits mRNA 5′ capping machinery to the nascent RNA, consistent with capping occurring co-transcriptionally [164,165,166]. Phosphoproteomic studies have shown CDK7 to target many other transcription-associated substrates, and inhibition of CDK7 in cells led to defects in splicing, cell-cycle regulation, and RNA processing [43,165,166,167,168]. Experiments show that CDK7 inhibition alters Pol II occupancy across genes and transcribed RNA in patterns that suggest defects not only in initiation, but promoter proximal pausing and termination [169,170]. Taken together, these results showcase the critical role that the CDK7 kinase plays not only during initiation but also in multiple stages of transcription and co-transcriptional RNA processing.

## 5. Early Transcription

### 5.1. Promoter Escape

During early transcription, transcribing complexes undergo numerous rearrangements as initiation complexes transform into elongation complexes and Pol II begins to move away from the start site of transcription. This stage of the transcription reaction, referred to a promoter escape, is less well defined than other stages. The complexity of promoter escape lies in the wide array of structural transformations that must occur as Pol II transitions from an initiation complex into an elongation complex. Protein–protein and protein–nucleic acid contacts formed in PICs must be broken, while other contacts are established, and several GTFs are thought to release from the transcribing complex [171,172]. In addition, the melted region of DNA expands then collapses to the size maintained during elongation [173]. Due to the transient nature of promoter escape, this stage of the transcription reaction is difficult to assay in cells. Therefore, current understanding relies largely on structural and biochemical approaches. In vitro kinetic studies show the rate-limiting step of promoter escape is complete when an 8-nucleotide RNA is made [168], and the polymerase clears the core promoter via synthesis of the initial 20–30 nucleotides of mRNA [171].

During promoter escape, early transcribing complexes have two fates: continue transcribing toward productive elongation or abortive transcription. Abortive transcription occurs when Pol II halts transcription after synthesizing a short mRNA product and releases from the template DNA. Research has shown that only a small fraction (~5–20%) of all PICs that form in vitro continue on to productive elongation [174,175,176]. Within cells, evidence suggests that less than 15% of Pol II molecules interacting with genes reach the initiation step, and an even smaller fraction proceed to elongation. This leads to a low percentage of Pol II/DNA interactions producing an mRNA transcript [60,63,177,178]. The biological significance of having such a small population of active complexes is a topic of great interest and requires distinguishing between a small population of productive complexes and the population of inactive complexes/interactions. While the cause of this heterogeneity in activity is unknown, it is likely that only the complexes that successfully complete all transformations that occur during promoter escape can proceed to elongation, which prevents improperly assembled complexes from continuing to elongate the transcript. These transformations include conformational changes among Pol II and the GTFs, correct positioning of the DNA in the Pol II cleft, and recruitment of elongation factors and other co-transcriptional machinery, among others.

During the transition to an elongation complex, Pol II needs to break contacts with promoter-bound GTFs as it transcribes away from the TSS. In vitro experiments show that TFIIB, TFIIE, and TFIIH release at points during early transcription [57,147]; TFIIF remains bound to Pol II during promoter escape but is not stably associated with Pol II later in elongation [123,132,133]. Studies suggest that TFIIF facilitates promoter escape by suppressing abortive transcription, restoring stalled Pol II complexes, and enhancing the effects of other positive elongation factors, such as TFIIS [179]. Biochemical studies suggest that TFIID remains bound at the core promoter, and TFIIB can re-associate with TFIID [133,180]. This may serve as a transcriptional “memory” to mark actively transcribed genes as cells progress through the cell cycle. For example, as cells pause transcription upon entry into mitosis, chromatin becomes compacted, and Pol II is removed from the genome; however, many sites still show high levels of TFIIB and TFIID occupancy through this period of gene silencing [181].

### 5.2. Promoter Proximal Pausing

The next major regulatory checkpoint is promoter proximal pausing (PPP), which occurs at nearly all Pol II-transcribed metazoan genes around 30–100 bases downstream of the TSS [182,183,184]. PPP is a highly regulated event in which Pol II pauses transcription and can either undergo premature termination or release into the gene body to proceed through transcript elongation. Multiple biological systems utilize PPP regulation to achieve temporal transcriptional control, including the immune response [185], hormone signaling [186,187], and early development [188,189], underlining the broad physiological relevance of this phenomenon.

**Figure 3:**
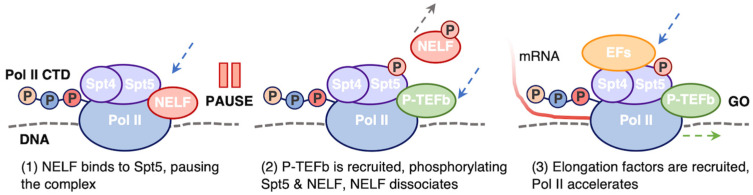
Promoter proximal pausing mechanism of Pol II. Around 30–100 bases downstream of the TSS, Pol II pauses transcription due to the recruitment (blue arrow) of NELF and DSIF (Spt4 and Spt5) (**1**). Pausing is released when P-TEFb is recruited (blue arrow), Spt5 and NELF are phosphorylated by the CDK9 subunit of P-TEFb, and NELF dissociates (gray arrow) (**2**). As Pol II transitions to productive elongation, elongation factors (EFs) are recruited (blue arrow), thereby increasing Pol II elongation efficiency (green arrow) (**3**).

From a mechanistic perspective, PPP is predominantly caused by binding of DRB Sensitivity Inducing Factor (DSIF) and Negative Elongation Factor (NELF) [190] (Figure 3). As Pol II escapes the promoter region, DSIF is recruited. DSIF is a heterodimer of Spt4 and Spt5 subunits, the latter of which contacts Pol II near the mRNA exit channel and facilitates 5′ end capping of the mRNA [191,192]. The Spt5 subunit of DSIF contacts the same Pol II interface as TFIIE; therefore, DSIF may be recruited as TFIIE is released [193,194]. DSIF binds to NELF, or Negative Elongation factor, which stabilizes the paused complex and extends the lifetime of the paused state [190,195,196,197]. Pause release occurs with the recruitment of positive transcription elongation factor b (P-TEFb), which contains the CDK9 kinase. CDK9 phosphorylates both NELF and the Spt5 subunit of DSIF, triggering dissociation of NELF [196,198]. DSIF remains bound to the elongation complex after phosphorylation, stimulating Pol II elongation and recruiting other elongation factors [183]. In addition to phosphorylating Spt5 in DSIF, CDK9 also phosphorylates other elongation factors, chromatin modifiers, RNA processing factors, and Ser2 on the Pol II CTD, a known mark of active elongation [199,200]. Therefore, P-TEFb broadly encourages productive elongation and pause release through multiple phosphorylation targets in the elongation complex. Promoter-proximally paused complexes can undergo premature termination as opposed to release into productive elongation through mechanisms involving the multi-subunit Integrator complex [201]. The ratio of complexes released into elongation versus prematurely terminating and the rate of turnover of paused Pol II complexes are open areas of investigation and likely provide an additional layer of regulation for specific genes within different cell types.

Ongoing research studies have used inhibitors to probe the regulation and biological function of this important regulatory step in early transcription. Studies with inhibitors of CDK9 have shown that PPP is essential for productive transcription [202,203]. It is possible that PPP occurs to allow for conformational changes within the early elongation complex to take place, leading to higher stability of the Pol II transcription complex. Pausing may also allow for the recruitment of the full suite of elongation factors necessary for Pol II to transition to productive elongation. Additionally, PPP adds yet another layer of transcriptional regulation in response to changing cellular conditions: Pol II may undergo continuous cycles of PIC assembly, elongation to the PPP site, pausing, and termination until a signal allows for the transition to productive elongation [197].

## 6. Elongation

After release from promoter proximal pausing, Pol II transitions to an elongation complex that can productively synthesize RNA. The elongation stage of Pol II transcription, and its regulation, is complex due to the involvement of a multitude of cofactors, regulators, chromatin interactions, and co-transcriptional processes. Elongation by Pol II is reviewed in detail elsewhere [204,205], with key points emphasized here. During elongation, mRNA is synthesized at speeds > 2000 nucleotides per minute, as measured in cells after releasing Pol II from a drug-induced pause [202]. This rate drops to 300 nucleotides per minute using purified Pol II in an in vitro system [206], emphasizing the importance of elongation factors interacting with Pol II. Elongation factors include Poly (ADP-ribose) Polymerases (PARPs), elongation factor for RNA Pol II (ELL), TFIIS, elongin A, DSIF, and Spt6, among others [207]. Some of these elongation factors can also form subcomplexes of different compositions that work together to modulate Pol II elongation, such as the super elongation complex (SEC) consisting of P-TEFb, ELL proteins, and AFF family members [208,209].

Another important complex that co-regulates Pol II elongation activity is the Polymerase-associated factor 1 Complex, or Paf1C [210,211]. This complex of six subunits in humans (five subunits in some organisms) is highly conserved across different levels of eukaryotes. Loss of Paf1C from mammalian cells causes accumulation of Pol II on gene bodies and slower elongation rates, while in vitro studies have shown a direct stimulatory role of Paf1C on elongation efficiency [211]. Paf1C interacts directly with the phosphorylated Pol II CTD tail and DSIF in the elongation complex [212,213,214,215]. Paf1C is recruited after the CDK9 kinase of P-TEFb phosphorylates NELF and DSIF, which leads to NELF dissociation, enabling Paf1C binding to DSIF. Some studies suggest that Paf1C and CDK9 share a mutual dependence for recruitment to active chromatin, thus implicating Paf1C in promoter proximal pausing. However, the exact role that Paf1C plays in pausing regulation remains unclear and is an ongoing point of investigation [216,217,218,219]. Paf1C has also been shown to associate with a myriad of other proteins, including gene-specific transcription factors and factors associated with developmental signaling pathways [220,221]. Due to its interactions with a vast number of factors, Paf1C is an important regulatory point for Pol II elongation.

During elongation, Pol II must transcribe through nucleosomal DNA, which is completed with the help of chromatin remodelers such as Chd1 and FACT [222,223]. As Pol II transcribes through the genome at high speed, these factors rearrange histone complexes ahead of the polymerase and replace histones behind the elongation complex [4,224]. Recent evidence also suggests that FACT recycles nucleosomes to maintain epigenetic modifications on the histone tails, thus maintaining the chromatin state through rounds of transcription [225]. Studies have implicated Paf1C in chromatin modifications and epigenetic control through direct contacts with related factors, including FACT [226,227] and Chd1 [228,229]. Additionally, Paf1C has been associated with the maintenance of several histone marks for active chromatin, further emphasizing its role as an important regulator of Pol II productive elongation [229,230,231,232].

Throughout the elongation and ultimately termination phases of transcription, the phosphorylation state of the Pol II CTD changes, which is thought to help recruit and regulate elongation factors and co-transcriptional machinery. For example, Ser2 phosphorylation increases as Pol II moves throughout the gene body, which signals the entry of Pol II into the elongation phase and facilitates association of elongation, termination, splicing, and nuclear export factors with the transcribing complex [36,233,234,235,236,237]. Tyr1 phosphorylation, which peaks at the promoter proximal pause, is present within the gene body before falling near the 3′ end of the gene. In yeast, it has been shown that Tyr1 phosphorylation impairs recruitment of termination factors to prevent premature termination, thereby acting to promote elongation [238]. Accordingly, dephosphorylation of Tyr1 is also important for proper termination in yeast [239]. Consistent with this, in human cells, mutating the majority of Tyr1 residues to phenylalanines in the Pol II CTD caused a genome-wide termination defect [240]. In both yeast and humans, Thr4 follows a similar phosphorylation profile to Ser2, becoming phosphorylated throughout the gene body and peaking at the 3′ end of genes [23]. ChIP-seq studies in human cells revealed that Thr4 is essential for productive elongation and that mutation of Thr4 leads to a genome-wide elongation defect [241]. Moreover, in human cells Thr4 phosphorylation is thought to be important for 3′ end processing of nascent transcripts [38].

## 7. Termination

**Figure 4:**
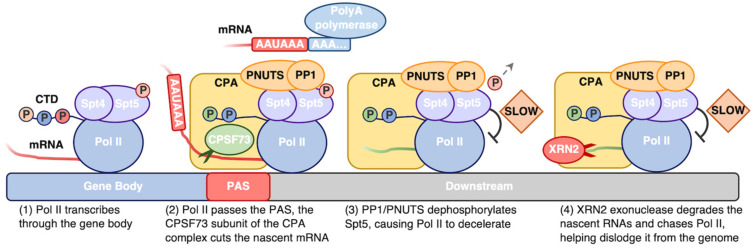
Pol II requires a complex network of factors to facilitate termination. Pol II transcribes through the gene body at over 2 kilobases per minute (**1**). Subunits in the CPA complex recognize the PAS in the transcript RNA, along with other regulatory sequences. The CPSF73 endonuclease subunit cleaves the nascent RNA to generate the 3′ end of the mRNA, which undergoes further processing such as addition of up to several hundred adenosine residues to the 3′ end of the cleaved mRNA by polyA polymerase (**2**). PP1/PNUTS dephosphorylates the Spt5 subunit of DSIF (gray arrow), which causes Pol II to decelerate (**3**). The XRN2 exonuclease binds to the 5′ end of the nascent transcript, digesting it until it until Pol II is dislodged from the genome (**4**).

The final stage in a single round of transcription is termination. This step is described briefly below (Figure 4) and is reviewed more thoroughly elsewhere in the literature [242,243,244,245]. Elongation proceeds until Pol II transcribes through the polyadenylation site (PAS; AAUAAA in human mRNA transcripts), which signals for transcription termination. The polymerase slows down during this phase of the reaction, which is facilitated by dephosphorylation of the Spt5 subunit of DSIF by the PP1–PNUTS phosphatase complex [246]. The cleavage and polyadenylation complex (CPA) associates with Pol II, likely due to CTD interactions. The CPA, constituting the core of the termination machinery, contains an array of multi-subunit factors that recognize the PAS and other sequences in the mRNA. This complex includes an endonuclease (CPSF73) that cleaves the nascent mRNA to generate the 3′ end of the mRNA, and a polyA polymerase that attaches up to several hundred adenosine residues to the 3′ end [242,243,244,245]. Polyadenylation of the 3′ end protects the mRNA from exonuclease degradation and aids in nuclear export and translation [247]. Some genes contain multiple PAS sequences, allowing for alternative termination and polyadenylation of the mRNA product akin to alternative splicing variants [248]. Paf1C is also involved in termination by regulating co-transcriptional processes, including the cleavage and polyadenylation machinery [249,250,251], as well as post-transcriptional processes, including mRNA export machinery [250,251,252].

Cleavage of the nascent mRNA by CPSF73 occurs just downstream of the polyA site, which facilitates termination through two mechanisms that are not mutually exclusive: the “allosteric” model and the “torpedo” model [244]. The former model suggests that allosteric changes occur within the elongation complex and/or the nascent mRNA, which prompt transcribing Pol II to release the DNA template and its mRNA product [253,254]. In the torpedo model, after cleavage of the nascent mRNA, the exonuclease Xrn2 accesses the 5′ end of the nascent RNA strand behind the still transcribing Pol II and races to catch and displace Pol II, resulting in termination [246]. It is likely both of these models contribute to regulation of termination [255]. After dissociation, Pol II is then free to assemble into other transcription complexes, and the new mRNA product is exported to the cytosol for further processing and translation.

## 8. Summary and Future Perspectives

The complexity of the Pol II transcription system has challenged researchers for decades as they work to understand the many layers of regulation, protein factors involved, and co-transcriptional processes. The field has elucidated general factors and fundamental mechanisms at each of the major stages of transcription (PIC assembly, initiation, promoter escape, PPP, elongation, and termination), but there are still many unanswered questions left to investigate. Structural biologists are achieving high-resolution structures of Pol II complexes and their interacting partners, which will continue to provide mechanistic insight into how these large and flexible complexes function. This will be complemented by studies using imaging technologies (e.g., biochemical single-molecule and live-cell single-particle microscopy) to reveal new modes of regulation by dynamically tracking the GTFs, ultimately through the stages of transcription in real-time. Research dedicated to understanding intergenic regions of the genome will uncover more regulatory mechanisms for gene expression, including enhancers and long non-coding RNAs. Advancements in sequencing methods, bioinformatics, proteomics, and computational biology will illustrate how Pol II works with various partners to regulate gene expression, and how the genome itself is structured to globally modulate the Pol II system. The immense complexity of transcription and the human genome provides not only a great challenge for researchers but also incredible reward in understanding how the two are intricately connected and balanced.

## Figures and Tables

**Table 1:** Summary of the general transcription factors and RNA polymerase II.

Protein	Subunits	Size (kDa)	Main Binding Partners	Function
TFIID	TBP, 13 TAFs	~1300	promoter, Pol II	Nucleates PIC assembly by binding multiple core promoter elements
TFIIA	TFIIAα, TFIIAβ, TFIIAɣ	35, 19, and 12	TBP, TFIID	Stabilizes the TFIID-DNA interaction; enhances the effects of transcriptional co-activators
TFIIB	TFIIB	33	promoter, TBP, Pol II	Helps to define the start site of transcription and orient Pol II in the proper direction
TFIIF	RAP30, RAP74	30 and 74	promoter, Pol II, GTFs	Guides Pol II to the PIC and facilitates elongation
Pol II	Rpb1–Rpb12	~514	promoter, all GTFs	Catalyzes RNA synthesis; phosphorylation of the CTD tail of Rpb1 serves a regulatory role
TFIIE	TFIIEα, TFIIEβ	56 and 34	promoter, TFIIH, Pol II, TFIIF	Recruits TFIIH to the PIC; stimulates enzymatic activities of TFIIH; stabilizes the open DNA conformation
TFIIH	Core domain: XPD, XBP, p62, p52, p44, p34, p8; CAK domain: CDK7, MAT1, cyclin H	~500	downstream DNA, TFIIE, Pol II	CDK7 kinase phosphorylates the CTD; ATP-dependent XPB translocase opens the promoter DNA

## References

[1] Richter W.F., Nayak S., Iwasa J., Taatjes D.J. (2022). The Mediator Complex as a Master Regulator of Transcription by RNA Polymerase II. Nat. Rev. Mol. Cell Biol..

[2] Soutourina J. (2018). Transcription Regulation by the Mediator Complex. Nat. Rev. Mol. Cell Biol..

[3] Li B., Carey M., Workman J.L. (2007). The Role of Chromatin during Transcription. Cell.

[4] Kujirai T., Kurumizaka H. (2020). Transcription through the Nucleosome. Curr. Opin. Struct. Biol..

[5] Lim B., Levine M.S. (2021). Enhancer-Promoter Communication: Hubs or Loops?. Curr. Opin. Genet. Dev..

[6] Kadonaga J.T. (2004). Regulation of RNA Polymerase II Transcription by Sequence-Specific DNA Binding Factors. Cell.

[7] Malik S., Roeder R.G. (2023). Regulation of the RNA Polymerase II Pre-Initiation Complex by Its Associated Coactivators. Nat. Rev. Genet..

[8] Chen H., Pugh B.F. (2021). What Do Transcription Factors Interact with?. J. Mol. Biol..

[9] Cramer P., Armache K.-J., Baumli S., Benkert S., Brueckner F., Buchen C., Damsma G.E., Dengl S., Geiger S.R., Jasiak A.J. (2008). Structure of Eukaryotic RNA Polymerases. Annu. Rev. Biophys..

[10] Vannini A., Cramer P. (2012). Conservation between the RNA Polymerase I, II, and III Transcription Initiation Machineries. Mol. Cell.

[11] Li Y., Huang J., Zhu J., Bao L., Wang H., Jiang Y., Tian K., Wang R., Zheng H., Duan W. (2022). Targeted Protein Degradation Reveals RNA Pol II Heterogeneity and Functional Diversity. Mol. Cell.

[12] Osman S., Cramer P. (2020). Structural Biology of RNA Polymerase II Transcription: 20 Years on. Annu. Rev. Cell Dev. Biol..

[13] Schier A.C., Taatjes D.J. (2020). Structure and Mechanism of the RNA Polymerase II Transcription Machinery. Genes Dev..

[14] Aibara S., Schilbach S., Cramer P. (2021). Structures of Mammalian RNA Polymerase II Pre-Initiation Complexes. Nature.

[15] Schier A.C., Taatjes D.J. (2021). Everything at Once: Cryo-EM Yields Remarkable Insights into Human RNA Polymerase II Transcription. Nat. Struct. Mol. Biol..

[16] Gnatt A.L., Cramer P., Fu J., Bushnell D.A., Kornberg R.D. (2001). Structural Basis of Transcription: An RNA Polymerase II Elongation Complex at 3.3 Å Resolution. Science.

[17] Hahn S. (2004). Structure and Mechanism of the RNA Polymerase II Transcription Machinery. Nat. Struct. Mol. Biol..

[18] Armache K.-J., Kettenberger H., Cramer P. (2003). Architecture of Initiation-Competent 12-Subunit RNA Polymerase II. Proc. Natl. Acad. Sci. USA.

[19] Bushnell D.A., Westover K.D., Davis R.E., Kornberg R.D. (2004). Structural Basis of Transcription: An RNA Polymerase II–TFIIB Cocrystal at 4.5 Angstroms. Science.

[20] Dienemann C., Schwalb B., Schilbach S., Cramer P. (2019). Promoter Distortion and Opening in the RNA Polymerase II Cleft. Mol. Cell.

[21] Bernecky C., Herzog F., Baumeister W., Plitzko J.M., Cramer P. (2016). Structure of Transcribing Mammalian RNA Polymerase II. Nature.

[22] Lyons D.E., McMahon S., Ott M. (2020). A Combinatorial View of Old and New RNA Polymerase II Modifications. Transcription.

[23] Harlen K.M., Churchman L.S. (2017). The Code and beyond: Transcription Regulation by the RNA Polymerase II Carboxy-Terminal Domain. Nat. Rev. Mol. Cell Biol..

[24] Liu P., Kenney J.M., Stiller J.W., Greenleaf A.L. (2010). Genetic Organization, Length Conservation, and Evolution of RNA Polymerase II Carboxyl-Terminal Domain. Mol. Biol. Evol..

[25] Zehring W.A., Lee J.M., Weeks J.R., Jokerst R.S., Greenleaf A.L. (1988). The C-Terminal Repeat Domain of RNA Polymerase II Largest Subunit Is Essential in Vivo but Is Not Required for Accurate Transcription Initiation in Vitro. Proc. Natl. Acad. Sci. USA.

[26] Kim W.Y., Dahmus M.E. (1989). The Major Late Promoter of Adenovirus-2 Is Accurately Transcribed by RNA Polymerases IIO, IIA, and IIB. J. Biol. Chem..

[27] Gerber H.-P., Hagmann M., Seipel K., Georgiev O., West M.A.L., Litingtung Y., Schaffner W., Corden J.L. (1995). RNA Polymerase II C-Terminal Domain Required for Enhancer-Driven Transcription. Nature.

[28] Yahia Y., Pigeot A., El Aabidine A.Z., Shah N., Karasu N., Forné I., Krebs S., Blum H., Esnault C., Sexton T. (2023). RNA Polymerase II CTD Is Dispensable for Transcription and Required for Termination in Human Cells. EMBO Rep..

[29] Garg A., Sanchez A.M., Schwer B., Shuman S. (2021). Transcriptional Profiling of Fission Yeast RNA Polymerase II CTD Mutants. RNA.

[30] Singh N., Asalam M., Ansari M.O., Gerasimova N.S., Studitsky V.M., Akhtar M.S. (2022). Transcription by RNA Polymerase II and the CTD-Chromatin Crosstalk. Biochem. Biophys. Res. Commun..

[31] Maita H., Nakagawa S. (2020). What Is the Switch for Coupling Transcription and Splicing? RNA Polymerase II C-terminal Domain Phosphorylation, Phase Separation and Beyond. WIREs RNA.

[32] Jeronimo C., Bataille A.R., Robert F. (2013). The Writers, Readers, and Functions of the RNA Polymerase II C-Terminal Domain Code. Chem. Rev..

[33] Davidson L., Muniz L., West S. (2014). 3′ End Formation of Pre-mRNA and Phosphorylation of Ser2 on the RNA Polymerase II CTD Are Reciprocally Coupled in Human Cells. Genes Dev..

[34] Nojima T., Gomes T., Grosso A.R.F., Kimura H., Dye M.J., Dhir S., Carmo-Fonseca M., Proudfoot N.J. (2015). Mammalian NET-Seq Reveals Genome-Wide Nascent Transcription Coupled to RNA Processing. Cell.

[35] Ahn S.H., Kim M., Buratowski S. (2004). Phosphorylation of Serine 2 within the RNA Polymerase II C-Terminal Domain Couples Transcription and 3′ End Processing. Mol. Cell.

[36] Gu B., Eick D., Bensaude O. (2013). CTD Serine-2 Plays a Critical Role in Splicing and Termination Factor Recruitment to RNA Polymerase II in Vivo. Nucleic Acids Res..

[37] Heidemann M., Eick D. (2012). Tyrosine-1 and Threonine-4 Phosphorylation Marks Complete the RNA Polymerase II CTD Phospho-Code. RNA Biol..

[38] Yurko N.M., Manley J.L. (2018). The RNA Polymerase II CTD “Orphan” Residues: Emerging Insights into the Functions of Tyr-1, Thr-4, and Ser-7. Transcription.

[39] Thomas M.C., Chiang C.-M. (2006). The General Transcription Machinery and General Cofactors. Crit. Rev. Biochem. Mol. Biol..

[40] Luse D.S. (2014). The RNA Polymerase II Preinitiation Complex: Through What Pathway Is the Complex Assembled?. Transcription.

[41] Flores O., Lu H., Reinberg D. (1992). Factors Involved in Specific Transcription by Mammalian RNA Polymerase II. Identification and Characterization of Factor IIH. J. Biol. Chem..

[42] Buratowski S., Hahn S., Guarente L., Sharp P.A. (1989). Five Intermediate Complexes in Transcription Initiation by RNA Polymerase II. Cell.

[43] Ossipow V., Tassan J.-P., Nigg E.A., Schibler U. (1995). A Mammalian RNA Polymerase II Holoenzyme Containing All Components Required for Promoter-Specific Transcription Initiation. Cell.

[44] Wu S.-Y., Chiang C.-M. (1998). Properties of PC4 and an RNA Polymerase II Complex in Directing Activated and Basal Transcription in Vitro. J. Biol. Chem..

[45] Cramer P. (2019). Organization and Regulation of Gene Transcription. Nature.

[46] Hantsche M., Cramer P. (2017). Conserved RNA Polymerase II Initiation Complex Structure. Curr. Opin. Struct. Biol..

[47] Hwang D.-W., Maekiniemi A., Singer R.H., Sato H. (2024). Real-Time Single-Molecule Imaging of Transcriptional Regulatory Networks in Living Cells. Nat. Rev. Genet..

[48] Wagh K., Stavreva D.A., Upadhyaya A., Hager G.L. (2023). Transcription Factor Dynamics: One Molecule at a Time. Annu. Rev. Cell Dev. Biol..

[49] Wang Z., Deng W. (2022). Dynamic Transcription Regulation at the Single-Molecule Level. Dev. Biol..

[50] Lionnet T., Wu C. (2021). Single-Molecule Tracking of Transcription Protein Dynamics in Living Cells: Seeing Is Believing, but What Are We Seeing?. Curr. Opin. Genet. Dev..

[51] Chen J., Zhang Z., Li L., Chen B.-C., Revyakin A., Hajj B., Legant W., Dahan M., Lionnet T., Betzig E. (2014). Single-Molecule Dynamics of Enhanceosome Assembly in Embryonic Stem Cells. Cell.

[52] Hipp L., Beer J., Kuchler O., Reisser M., Sinske D., Michaelis J., Gebhardt J.C.M., Knöll B. (2019). Single-Molecule Imaging of the Transcription Factor SRF Reveals Prolonged Chromatin-Binding Kinetics upon Cell Stimulation. Proc. Natl. Acad. Sci. USA.

[53] Hansen A.S., Amitai A., Cattoglio C., Tjian R., Darzacq X. (2020). Guided Nuclear Exploration Increases CTCF Target Search Efficiency. Nat. Chem. Biol..

[54] Izeddin I., Récamier V., Bosanac L., Cissé I.I., Boudarene L., Dugast-Darzacq C., Proux F., Bénichou O., Voituriez R., Bensaude O. (2014). Single-Molecule Tracking in Live Cells Reveals Distinct Target-Search Strategies of Transcription Factors in the Nucleus. eLife.

[55] Paakinaho V., Presman D.M., Ball D.A., Johnson T.A., Schiltz R.L., Levitt P., Mazza D., Morisaki T., Karpova T.S., Hager G.L. (2017). Single-Molecule Analysis of Steroid Receptor and Cofactor Action in Living Cells. Nat. Commun..

[56] Zhang Z., English B.P., Grimm J.B., Kazane S.A., Hu W., Tsai A., Inouye C., You C., Piehler J., Schultz P.G. (2016). Rapid Dynamics of General Transcription Factor TFIIB Binding during Preinitiation Complex Assembly Revealed by Single-Molecule Analysis. Genes Dev..

[57] Ly E., Powell A.E., Goodrich J.A., Kugel J.F. (2020). Release of Human TFIIB from Actively Transcribing Complexes Is Triggered upon Synthesis of 7- and 9-Nt RNAs. J. Mol. Biol..

[58] Baek I., Friedman L.J., Gelles J., Buratowski S. (2021). Single-Molecule Studies Reveal Branched Pathways for Activator-Dependent Assembly of RNA Polymerase II Pre-Initiation Complexes. Mol. Cell.

[59] Hou T.Y., Kraus W.L. (2021). Come One, Come All? Re-Evaluating RNA Polymerase II Pre-Initiation Complex Assembly Using Single-Molecule Microscopy. Mol. Cell.

[60] Nguyen V.Q., Ranjan A., Liu S., Tang X., Ling Y.H., Wisniewski J., Mizuguchi G., Li K.Y., Jou V., Zheng Q. (2021). Spatiotemporal Coordination of Transcription Preinitiation Complex Assembly in Live Cells. Mol. Cell.

[61] Li J., Dong A., Saydaminova K., Chang H., Wang G., Ochiai H., Yamamoto T., Pertsinidis A. (2019). Single-Molecule Nanoscopy Elucidates RNA Polymerase II Transcription at Single Genes in Live Cells. Cell.

[62] Stasevich T.J., Hayashi-Takanaka Y., Sato Y., Maehara K., Ohkawa Y., Sakata-Sogawa K., Tokunaga M., Nagase T., Nozaki N., McNally J.G. (2014). Regulation of RNA Polymerase II Activation by Histone Acetylation in Single Living Cells. Nature.

[63] Steurer B., Janssens R.C., Geverts B., Geijer M.E., Wienholz F., Theil A.F., Chang J., Dealy S., Pothof J., Van Cappellen W.A. (2018). Live-Cell Analysis of Endogenous GFP-RPB1 Uncovers Rapid Turnover of Initiating and Promoter-Paused RNA Polymerase II. Proc. Natl. Acad. Sci. USA.

[64] Tunnacliffe E., Chubb J.R. (2020). What Is a Transcriptional Burst?. Trends Genet..

[65] Cisse I.I., Izeddin I., Causse S.Z., Boudarene L., Senecal A., Muresan L., Dugast-Darzacq C., Hajj B., Dahan M., Darzacq X. (2013). Real-Time Dynamics of RNA Polymerase II Clustering in Live Human Cells. Science.

[66] Tantale K., Mueller F., Kozulic-Pirher A., Lesne A., Victor J.-M., Robert M.-C., Capozi S., Chouaib R., Bäcker V., Mateos-Langerak J. (2016). A Single-Molecule View of Transcription Reveals Convoys of RNA Polymerases and Multi-Scale Bursting. Nat. Commun..

[67] Sabari B.R., Dall’Agnese A., Boija A., Klein I.A., Coffey E.L., Shrinivas K., Abraham B.J., Hannett N.M., Zamudio A.V., Manteiga J.C. (2018). Coactivator Condensation at Super-Enhancers Links Phase Separation and Gene Control. Science.

[68] Cho W.-K., Spille J.-H., Hecht M., Lee C., Li C., Grube V., Cisse I.I. (2018). Mediator and RNA Polymerase II Clusters Associate in Transcription-Dependent Condensates. Science.

[69] Du M., Stitzinger S.H., Spille J.-H., Cho W.-K., Lee C., Hijaz M., Quintana A., Cissé I.I. (2024). Direct Observation of a Condensate Effect on Super-Enhancer Controlled Gene Bursting. Cell.

[70] Palacio M., Taatjes D.J. (2022). Merging Established Mechanisms with New Insights: Condensates, Hubs, and the Regulation of RNA Polymerase II Transcription. J. Mol. Biol..

[71] Kimura H., Sato Y. (2022). Imaging Transcription Elongation Dynamics by New Technologies Unveils the Organization of Initiation and Elongation in Transcription Factories. Curr. Opin. Cell Biol..

[72] Luse D.S., Parida M., Spector B.M., Nilson K.A., Price D.H. (2020). A Unified View of the Sequence and Functional Organization of the Human RNA Polymerase II Promoter. Nucleic Acids Res..

[73] Starr D.B., Hawley D.K. (1991). TFIID Binds in the Minor Groove of the TATA Box. Cell.

[74] Santana J.F., Collins G.S., Parida M., Luse D.S., Price D.H. (2022). Differential Dependencies of Human RNA Polymerase II Promoters on TBP, TAF1, TFIIB and XPB. Nucleic Acids Res..

[75] Nikolov D.B., Chen H., Halay E.D., Hoffman A., Roeder R.G., Burley S.K. (1996). Crystal Structure of a Human TATA Box-Binding Protein/TATA Element Complex. Proc. Natl. Acad. Sci. USA.

[76] Starr B.D., Hoopes B.C., Hawley D.K. (1995). DNA Bending Is an Important Component of Site-Specific Recognition by the TATA Binding Protein. J. Mol. Biol..

[77] White R., Jackson S. (1992). The TATA-Binding Protein: A Central Role in Transcription by RNA Polymerases I, II and III. Trends Genet..

[78] Chen X., Qi Y., Wu Z., Wang X., Li J., Zhao D., Hou H., Li Y., Yu Z., Liu W. (2021). Structural Insights into Preinitiation Complex Assembly on Core Promoters. Science.

[79] Gershenzon N.I., Ioshikhes I.P. (2005). Synergy of Human Pol II Core Promoter Elements Revealed by Statistical Sequence Analysis. Bioinformatics.

[80] Yang C., Bolotin E., Jiang T., Sladek F.M., Martinez E. (2007). Prevalence of the Initiator over the TATA Box in Human and Yeast Genes and Identification of DNA Motifs Enriched in Human TATA-Less Core Promoters. Gene.

[81] Suzuki Y., Tsunoda T., Sese J., Taira H., Mizushima-Sugano J., Hata H., Ota T., Isogai T., Tanaka T., Nakamura Y. (2001). Identification and Characterization of the Potential Promoter Regions of 1031 Kinds of Human Genes. Genome Res..

[82] Vo Ngoc L., Cassidy C.J., Huang C.Y., Duttke S.H.C., Kadonaga J.T. (2017). The Human Initiator Is a Distinct and Abundant Element That Is Precisely Positioned in Focused Core Promoters. Genes Dev..

[83] Zhang M.Q. (1998). Identification of Human Gene Core Promoters in Silico. Genome Res..

[84] Vo Ngoc L., Wang Y.-L., Kassavetis G.A., Kadonaga J.T. (2017). The Punctilious RNA Polymerase II Core Promoter. Genes Dev..

[85] Chalkley G.E. (1999). DNA Binding Site Selection by RNA Polymerase II TAFs: A TAFII250-TAFII150 Complex Recognizes the Initiator. EMBO J..

[86] Dreos R., Sloutskin A., Malachi N., Ideses D., Bucher P., Juven-Gershon T. (2021). Computational Identification and Experimental Characterization of Preferred Downstream Positions in Human Core Promoters. PLOS Comput. Biol..

[87] Vo Ngoc L., Huang C.Y., Cassidy C.J., Medrano C., Kadonaga J.T. (2020). Identification of the Human DPR Core Promoter Element Using Machine Learning. Nature.

[88] Theisen J.W.M., Lim C.Y., Kadonaga J.T. (2010). Three Key Subregions Contribute to the Function of the Downstream RNA Polymerase II Core Promoter. Mol. Cell. Biol..

[89] Louder R.K., He Y., López-Blanco J.R., Fang J., Chacón P., Nogales E. (2016). Structure of Promoter-Bound TFIID and Model of Human Pre-Initiation Complex Assembly. Nature.

[90] Joo Y.J., Ficarro S.B., Soares L.M., Chun Y., Marto J.A., Buratowski S. (2017). Downstream Promoter Interactions of TFIID TAFs Facilitate Transcription Reinitiation. Genes Dev..

[91] Nogales E., Louder R.K., He Y. (2017). Structural Insights into the Eukaryotic Transcription Initiation Machinery. Annu. Rev. Biophys..

[92] Fant C.B., Levandowski C.B., Gupta K., Maas Z.L., Moir J., Rubin J.D., Sawyer A., Esbin M.N., Rimel J.K., Luyties O. (2020). TFIID Enables RNA Polymerase II Promoter-Proximal Pausing. Mol. Cell.

[93] Sloutskin A., Shir-Shapira H., Freiman R.N., Juven-Gershon T. (2021). The Core Promoter Is a Regulatory Hub for Developmental Gene Expression. Front. Cell Dev. Biol..

[94] Haberle V., Stark A. (2018). Eukaryotic Core Promoters and the Functional Basis of Transcription Initiation. Nat. Rev. Mol. Cell Biol..

[95] Bernardini A., Hollinger C., Willgenss D., Müller F., Devys D., Tora L. (2023). Transcription Factor IID Parks and Drives Preinitiation Complexes at Sharp or Broad Promoters. Trends Biochem. Sci..

[96] Serebreni L., Pleyer L., Haberle V., Hendy O., Vlasova A., Loubiere V., Nemčko F., Bergauer K., Roitinger E., Mechtler K. (2023). Functionally Distinct Promoter Classes Initiate Transcription via Different Mechanisms Reflected in Focused versus Dispersed Initiation Patterns. EMBO J..

[97] Grzechnik P., Tan-Wong S.M., Proudfoot N.J. (2014). Terminate and Make a Loop: Regulation of Transcriptional Directionality. Trends Biochem. Sci..

[98] Bagchi D.N., Iyer V.R. (2016). The Determinants of Directionality in Transcriptional Initiation. Trends Genet..

[99] Seila A.C., Calabrese J.M., Levine S.S., Yeo G.W., Rahl P.B., Flynn R.A., Young R.A., Sharp P.A. (2008). Divergent Transcription from Active Promoters. Science.

[100] Preker P., Nielsen J., Kammler S., Lykke-Andersen S., Christensen M.S., Mapendano C.K., Schierup M.H., Jensen T.H. (2008). RNA Exosome Depletion Reveals Transcription Upstream of Active Human Promoters. Science.

[101] Core L.J., Waterfall J.J., Lis J.T. (2008). Nascent RNA Sequencing Reveals Widespread Pausing and Divergent Initiation at Human Promoters. Science.

[102] Andersson R., Gebhard C., Miguel-Escalada I., Hoof I., Bornholdt J., Boyd M., Chen Y., Zhao X., Schmidl C., Suzuki T. (2014). An Atlas of Active Enhancers across Human Cell Types and Tissues. Nature.

[103] Kim T.-K., Hemberg M., Gray J.M., Costa A.M., Bear D.M., Wu J., Harmin D.A., Laptewicz M., Barbara-Haley K., Kuersten S. (2010). Widespread Transcription at Neuronal Activity-Regulated Enhancers. Nature.

[104] He Y., Yan C., Fang J., Inouye C., Tjian R., Ivanov I., Nogales E. (2016). Near-Atomic Resolution Visualization of Human Transcription Promoter Opening. Nature.

[105] Høiby T., Zhou H., Mitsiou D.J., Stunnenberg H.G. (2007). A Facelift for the General Transcription Factor TFIIA. Biochim. Biophys. Acta BBA Gene Struct. Expr..

[106] Wang W., Gralla J.D., Carey M. (1992). The Acidic Activator GAL4-AH Can Stimulate Polymerase II Transcription by Promoting Assembly of a Closed Complex Requiring TFIID and TFIIA. Genes Dev..

[107] Lee D.K., DeJong J., Hashimoto S., Horikoshi M., Roeder R.G. (1992). TFIIA Induces Conformational Changes in TFIID via Interactions with the Basic Repeat. Mol. Cell. Biol..

[108] Imbalzano A.N., Zaret K.S., Kingston R.E. (1994). Transcription Factor (TF) IIB and TFIIA Can Independently Increase the Affinity of the TATA-Binding Protein for DNA. J. Biol. Chem..

[109] Coleman R.A., Taggart A.K.P., Burma S., Chicca J.J., Pugh B.F. (1999). TFIIA Regulates TBP and TFIID Dimers. Mol. Cell.

[110] Mitsiou D.J., Stunnenberg H.G. (2000). TAC, a TBP-sans-TAFs Complex Containing the Unprocessed TFIIAαβ Precursor and the TFIIAγ Subunit. Mol. Cell.

[111] Kang J.J., Auble D.T., Ranish J.A., Hahn S. (1995). Analysis of the Yeast Transcription Factor TFIIA: Distinct Functional Regions and a Polymerase II-Specific Role in Basal and Activated Transcription. Mol. Cell. Biol..

[112] Liu Q., Gabriel S.E., Roinick K.L., Ward R.D., Arndt K.M. (1999). Analysis of TFIIA Function In Vivo: Evidence for a Role in TATA-Binding Protein Recruitment and Gene-Specific Activation. Mol. Cell. Biol..

[113] Deng W., Roberts S.G.E. (2007). TFIIB and the Regulation of Transcription by RNA Polymerase II. Chromosoma.

[114] Deng W., Roberts S.G.E. (2005). A Core Promoter Element Downstream of the TATA Box That Is Recognized by TFIIB. Genes Dev..

[115] Lagrange T., Kapanidis A.N., Tang H., Reinberg D., Ebright R.H. (1998). New Core Promoter Element in RNA Polymerase II-Dependent Transcription: Sequence-Specific DNA Binding by Transcription Factor IIB. Genes Dev..

[116] Nikolov D.B., Chen H., Halay E.D., Usheva A.A., Hisatake K., Lee D.K., Roeder R.G., Burley S.K. (1995). Crystal Structure of a TFIIB–TBP–TATA-Element Ternary Complex. Nature.

[117] Orphanides G., Lagrange T., Reinberg D. (1996). The General Transcription Factors of RNA Polymerase II. Genes Dev..

[118] Pardee T.S., Bangur C.S., Ponticelli A.S. (1998). The N-Terminal Region of Yeast TFIIB Contains Two Adjacent Functional Domains Involved in Stable RNA Polymerase II Binding and Transcription Start Site Selection. J. Biol. Chem..

[119] Elsby L.M., Roberts S.G.E. (2004). The Role of TFIIB Conformation in Transcriptional Regulation. Biochem. Soc. Trans..

[120] Sainsbury S., Niesser J., Cramer P. (2013). Structure and Function of the Initially Transcribing RNA Polymerase II–TFIIB Complex. Nature.

[121] Flores O., Maldonado E., Reinberg D. (1989). Factors Involved in Specific Transcription by Mammalian RNA Polymerase II. Factors IIE and IIF Independently Interact with RNA Polymerase II. J. Biol. Chem..

[122] Flores O., Lu H., Killeen M., Greenblatt J., Burton Z.F., Reinberg D. (1991). The Small Subunit of Transcription Factor IIF Recruits RNA Polymerase II into the Preinitiation Complex. Proc. Natl. Acad. Sci. USA.

[123] Price D.H., Sluder A.E., Greenleaf A.L. (1989). Dynamic Interaction between a Drosophila Transcription Factor and RNA Polymerase II. Mol. Cell. Biol..

[124] Killeen M.T., Greenblatt J.F. (1992). The General Transcription Factor RAP30 Binds to RNA Polymerase II and Prevents It from Binding Nonspecifically to DNA. Mol. Cell Biol..

[125] Robert F., Forget D., Li J., Greenblatt J., Coulombe B. (1996). Localization of Subunits of Transcription Factors IIE and IIF Immediately Upstream of the Transcriptional Initiation Site of the Adenovirus Major Late Promoter. J. Biol. Chem..

[126] He Y., Fang J., Taatjes D.J., Nogales E. (2013). Structural Visualization of Key Steps in Human Transcription Initiation. Nature.

[127] Groft C.M., Uljon S.N., Wang R., Werner M.H. (1998). Structural Homology between the Rap30 DNA-Binding Domain and Linker Histone H5: Implications for Preinitiation Complex Assembly. Proc. Natl. Acad. Sci. USA.

[128] Robert F., Douziech M., Forget D., Egly J.-M., Greenblatt J., Burton Z.F., Coulombe B. (1998). Wrapping of Promoter DNA around the RNA Polymerase II Initiation Complex Induced by TFIIF. Mol. Cell.

[129] Shilatifard A., Conaway R.C., Conaway J.W. (2003). The RNA Polymerase II Elongation Complex. Annu. Rev. Biochem..

[130] Yan Q., Moreland R.J., Conaway J.W., Conaway R.C. (1999). Dual Roles for Transcription Factor IIF in Promoter Escape by RNA Polymerase II. J. Biol. Chem..

[131] Elmendorf B.J., Shilatifard A., Yan Q., Conaway J.W., Conaway R.C. (2001). Transcription Factors TFIIF, ELL, and Elongin Negatively Regulate SII-Induced Nascent Transcript Cleavage by Non-Arrested RNA Polymerase II Elongation Intermediates. J. Biol. Chem..

[132] Joo Y.J., Ficarro S.B., Chun Y., Marto J.A., Buratowski S. (2019). In Vitro Analysis of RNA Polymerase II Elongation Complex Dynamics. Genes Dev..

[133] Zawel L., Kumar K.P., Reinberg D. (1995). Recycling of the General Transcription Factors during RNA Polymerase II Transcription. Genes Dev..

[134] Cojocaru M., Jeronimo C., Forget D., Bouchard A., Bergeron D., Côte P., Poirier G.G., Greenblatt J., Coulombe B. (2008). Genomic Location of the Human RNA Polymerase II General Machinery: Evidence for a Role of TFIIF and Rpb7 at Both Early and Late Stages of Transcription. Biochem. J..

[135] Chen Y., Kokic G., Dienemann C., Dybkov O., Urlaub H., Cramer P. (2023). Structure of the Transcribing RNA Polymerase II–Elongin Complex. Nat. Struct. Mol. Biol..

[136] Chen Y., Cramer P. (2024). RNA Polymerase II Elongation Factors Use Conserved Regulatory Mechanisms. Curr. Opin. Struct. Biol..

[137] Ohkuma Y., Hashimoto S., Wang C.K., Horikoshi M., Roeder R.G. (1995). Analysis of the Role of TFIIE in Basal Transcription and TFIIH-Mediated Carboxy-Terminal Domain Phosphorylation through Structure-Function Studies of TFIIE-α. Mol. Cell. Biol..

[138] Ohkuma Y., Roeder R.G. (1994). Regulation of TFIIH ATPase and Kinase Activities by TFIIE during Active Initiation Complex Formation. Nature.

[139] Okamoto T., Yamamoto S., Watanabe Y., Ohta T., Hanaoka F., Roeder R.G., Ohkuma Y. (1998). Analysis of the Role of TFIIE in Transcriptional Regulation through Structure-Function Studies of the TFIIEβ Subunit. J. Biol. Chem..

[140] Serizawa H., Conaway J.W., Conaway R.C. (1994). An Oligomeric Form of the Large Subunit of Transcription Factor (TF) IIE Activates Phosphorylation of the RNA Polymerase II Carboxyl-Terminal Domain by TFIIH. J. Biol. Chem..

[141] Kuldell N.H., Buratowski S. (1997). Genetic Analysis of the Large Subunit of Yeast Transcription Factor IIE Reveals Two Regions with Distinct Functions. Mol. Cell. Biol..

[142] Miwa K., Kojima R., Obita T., Ohkuma Y., Tamura Y., Mizuguchi M. (2016). Crystal Structure of Human General Transcription Factor TFIIE at Atomic Resolution. J. Mol. Biol..

[143] Okuda M., Tanaka A., Arai Y., Satoh M., Okamura H., Nagadoi A., Hanaoka F., Ohkuma Y., Nishimura Y. (2004). A Novel Zinc Finger Structure in the Large Subunit of Human General Transcription Factor TFIIE. J. Biol. Chem..

[144] Yokomori K., Verrijzer C.P., Tjian R. (1998). An Interplay between TATA Box-Binding Protein and Transcription Factors IIE and IIA Modulates DNA Binding and Transcription. Proc. Natl. Acad. Sci. USA.

[145] Okuda M., Watanabe Y., Okamura H., Hanaoka F., Ohkuma Y., Nishimura Y. (2000). Structure of the Central Core Domain of TFIIEβ with a Novel Double-Stranded DNA-Binding Surface. EMBO J..

[146] Forget D., Langelier M.-F., Thérien C., Trinh V., Coulombe B. (2004). Photo-Cross-Linking of a Purified Preinitiation Complex Reveals Central Roles for the RNA Polymerase II Mobile Clamp and TFIIE in Initiation Mechanisms. Mol. Cell. Biol..

[147] Compe E., Genes C.M., Braun C., Coin F., Egly J.-M. (2019). TFIIE Orchestrates the Recruitment of the TFIIH Kinase Module at Promoter before Release during Transcription. Nat. Commun..

[148] Holstege F.C., van der Vliet P.C., Timmers H.T. (1996). Opening of an RNA Polymerase II Promoter Occurs in Two Distinct Steps and Requires the Basal Transcription Factors IIE and IIH. EMBO J..

[149] Maxon M.E., Goodrich J.A., Tjian R. (1994). Transcription Factor IIE Binds Preferentially to RNA Polymerase IIa and Recruits TFIIH: A Model for Promoter Clearance. Genes Dev..

[150] Watanabe T., Hayashi K., Tanaka A., Furumoto T., Hanaoka F., Ohkuma Y. (2003). The Carboxy Terminus of the Small Subunit of TFIIE Regulates the Transition from Transcription Initiation to Elongation by RNA Polymerase II. Mol. Cell. Biol..

[151] Nogales E., Greber B.J. (2019). High-Resolution Cryo-EM Structures of TFIIH and Their Functional Implications. Curr. Opin. Struct. Biol..

[152] Rimel J.K., Taatjes D.J. (2018). The Essential and Multifunctional TFIIH Complex. Protein Sci..

[153] Compe E., Egly J.-M. (2012). TFIIH: When Transcription Met DNA Repair. Nat. Rev. Mol. Cell Biol..

[154] Schaeffer L., Roy R., Humbert S., Moncollin V., Vermeulen W., Hoeijmakers J.H.J., Chambon P., Egly J.-M. (1993). DNA Repair Helicase: A Component of BTF2 (TFIIH) Basic Transcription Factor. Science.

[155] Svejstrup J.Q., Vichi P., Egly J.M. (1996). The Multiple Roles of Transcription/Repair Factor TFIIH. Trends Biochem. Sci..

[156] Coin F., Oksenych V., Mocquet V., Groh S., Blattner C., Egly J.M. (2008). Nucleotide Excision Repair Driven by the Dissociation of CAK from TFIIH. Mol. Cell.

[157] Wong K.H., Jin Y., Struhl K. (2014). TFIIH Phosphorylation of the Pol II CTD Stimulates Mediator Dissociation from the Preinitiation Complex and Promoter Escape. Mol. Cell.

[158] Fishburn J., Tomko E., Galburt E., Hahn S. (2015). Double-Stranded DNA Translocase Activity of Transcription Factor TFIIH and the Mechanism of RNA Polymerase II Open Complex Formation. Proc. Natl. Acad. Sci. USA.

[159] Plaschka C., Hantsche M., Dienemann C., Burzinski C., Plitzko J., Cramer P. (2016). Transcription Initiation Complex Structures Elucidate DNA Opening. Nature.

[160] Cramer P., Bushnell D.A., Kornberg R.D. (2001). Structural Basis of Transcription: RNA Polymerase II at 2.8 Ångstrom Resolution. Science.

[161] Unarta I.C., Goonetilleke E.C., Wang D., Huang X. (2023). Nucleotide Addition and Cleavage by RNA Polymerase II: Coordination of Two Catalytic Reactions Using a Single Active Site. J. Biol. Chem..

[162] Brueckner F., Cramer P. (2008). Structural Basis of Transcription Inhibition by α-Amanitin and Implications for RNA Polymerase II Translocation. Nat. Struct. Mol. Biol..

[163] Robinson P.J., Trnka M.J., Bushnell D.A., Davis R.E., Mattei P.-J., Burlingame A.L., Kornberg R.D. (2016). Structure of a Complete Mediator-RNA Polymerase II Pre-Initiation Complex. Cell.

[164] Cho E.-J., Takagi T., Moore C.R., Buratowski S. (1997). mRNA Capping Enzyme Is Recruited to the Transcription Complex by Phosphorylation of the RNA Polymerase II Carboxy-Terminal Domain. Genes Dev..

[165] Komarnitsky P., Cho E.-J., Buratowski S. (2000). Different Phosphorylated Forms of RNA Polymerase II and Associated mRNA Processing Factors during Transcription. Genes Dev..

[166] Pei Y., Hausmann S., Ho C.K., Schwer B., Shuman S. (2001). The Length, Phosphorylation State, and Primary Structure of the RNA Polymerase II Carboxyl-Terminal Domain Dictate Interactions with mRNA Capping Enzymes. J. Biol. Chem..

[167] Rimel J.K., Poss Z.C., Erickson B., Maas Z.L., Ebmeier C.C., Johnson J.L., Decker T.-M., Yaron T.M., Bradley M.J., Hamman K.B. (2020). Selective Inhibition of CDK7 Reveals High-Confidence Targets and New Models for TFIIH Function in Transcription. Genes Dev..

[168] Hieb A.R., Baran S., Goodrich J.A., Kugel J.F. (2006). An 8 Nt RNA Triggers a Rate-Limiting Shift of RNA Polymerase II Complexes into Elongation. EMBO J..

[169] Ebmeier C.C., Erickson B., Allen B.L., Allen M.A., Kim H., Fong N., Jacobsen J.R., Liang K., Shilatifard A., Dowell R.D. (2017). Human TFIIH Kinase CDK7 Regulates Transcription-Associated Chromatin Modifications. Cell Rep..

[170] Glover-Cutter K., Larochelle S., Erickson B., Zhang C., Shokat K., Fisher R.P., Bentley D.L. (2009). TFIIH-Associated Cdk7 Kinase Functions in Phosphorylation of C-Terminal Domain Ser7 Residues, Promoter-Proximal Pausing, and Termination by RNA Polymerase II. Mol. Cell. Biol..

[171] Luse D.S. (2013). Promoter Clearance by RNA Polymerase II. Biochim. Biophys. Acta BBA Gene Regul. Mech..

[172] Saunders A., Core L.J., Lis J.T. (2006). Breaking Barriers to Transcription Elongation. Nat. Rev. Mol. Cell Biol..

[173] Pal M., Ponticelli A.S., Luse D.S. (2005). The Role of the Transcription Bubble and TFIIB in Promoter Clearance by RNA Polymerase II. Mol. Cell.

[174] Kugel J.F., Goodrich J.A. (1998). Promoter Escape Limits the Rate of RNA Polymerase II Transcription and Is Enhanced by TFIIE, TFIIH, and ATP on Negatively Supercoiled DNA. Proc. Natl. Acad. Sci. USA.

[175] Horn A.E., Kugel J.F., Goodrich J.A. (2016). Single Molecule Microscopy Reveals Mechanistic Insight into RNA Polymerase II Preinitiation Complex Assembly and Transcriptional Activity. Nucleic Acids Res..

[176] Kamakaka R.T., Tyree C.M., Kadonaga J.T. (1991). Accurate and Efficient RNA Polymerase II Transcription with a Soluble Nuclear Fraction Derived from Drosophila Embryos. Proc. Natl. Acad. Sci. USA.

[177] Darzacq X., Shav-Tal Y., De Turris V., Brody Y., Shenoy S.M., Phair R.D., Singer R.H. (2007). In Vivo Dynamics of RNA Polymerase II Transcription. Nat. Struct. Mol. Biol..

[178] Forero-Quintero L.S., Raymond W., Handa T., Saxton M.N., Morisaki T., Kimura H., Bertrand E., Munsky B., Stasevich T.J. (2021). Live-Cell Imaging Reveals the Spatiotemporal Organization of Endogenous RNA Polymerase II Phosphorylation at a Single Gene. Nat. Commun..

[179] Pal S., Biswas D. (2023). Promoter-Proximal Regulation of Gene Transcription: Key Factors Involved and Emerging Role of General Transcription Factors in Assisting Productive Elongation. Gene.

[180] Yudkovsky N., Ranish J.A., Hahn S. (2000). A Transcription Reinitiation Intermediate That Is Stabilized by Activator. Nature.

[181] Christova R., Oelgeschläger T. (2002). Association of Human TFIID–Promoter Complexes with Silenced Mitotic Chromatin in Vivo. Nat. Cell Biol..

[182] Dollinger R., Gilmour D.S. (2021). Regulation of Promoter Proximal Pausing of RNA Polymerase II in Metazoans. J. Mol. Biol..

[183] Core L., Adelman K. (2019). Promoter-Proximal Pausing of RNA Polymerase II: A Nexus of Gene Regulation. Genes Dev..

[184] Adelman K., Lis J.T. (2012). Promoter-Proximal Pausing of RNA Polymerase II: Emerging Roles in Metazoans. Nat. Rev. Genet..

[185] Adelman K., Kennedy M.A., Nechaev S., Gilchrist D.A., Muse G.W., Chinenov Y., Rogatsky I. (2009). Immediate Mediators of the Inflammatory Response Are Poised for Gene Activation through RNA Polymerase II Stalling. Proc. Natl. Acad. Sci. USA.

[186] Gupte R., Muse G.W., Chinenov Y., Adelman K., Rogatsky I. (2013). Glucocorticoid Receptor Represses Proinflammatory Genes at Distinct Steps of the Transcription Cycle. Proc. Natl. Acad. Sci. USA.

[187] Hah N., Danko C.G., Core L., Waterfall J.J., Siepel A., Lis J.T., Kraus W.L. (2011). A Rapid, Extensive, and Transient Transcriptional Response to Estrogen Signaling in Breast Cancer Cells. Cell.

[188] Lagha M., Bothma J.P., Esposito E., Ng S., Stefanik L., Tsui C., Johnston J., Chen K., Gilmour D.S., Zeitlinger J. (2013). Paused Pol II Coordinates Tissue Morphogenesis in the Drosophila Embryo. Cell.

[189] Williams L.H., Fromm G., Gokey N.G., Henriques T., Muse G.W., Burkholder A., Fargo D.C., Hu G., Adelman K. (2015). Pausing of RNA Polymerase II Regulates Mammalian Developmental Potential through Control of Signaling Networks. Mol. Cell.

[190] Vos S.M., Farnung L., Urlaub H., Cramer P. (2018). Structure of Paused Transcription Complex Pol II–DSIF–NELF. Nature.

[191] Pei Y., Shuman S. (2002). Interactions between Fission Yeast mRNA Capping Enzymes and Elongation Factor Spt5. J. Biol. Chem..

[192] Wen Y., Shatkin A.J. (1999). Transcription Elongation Factor hSPT5 Stimulates mRNA Capping. Genes Dev..

[193] Schulz S., Gietl A., Smollett K., Tinnefeld P., Werner F., Grohmann D. (2016). TFE and Spt4/5 Open and Close the RNA Polymerase Clamp during the Transcription Cycle. Proc. Natl. Acad. Sci. USA.

[194] Larochelle S., Amat R., Glover-Cutter K., Sansó M., Zhang C., Allen J.J., Shokat K.M., Bentley D.L., Fisher R.P. (2012). Cyclin-Dependent Kinase Control of the Initiation-to-Elongation Switch of RNA Polymerase II. Nat. Struct. Mol. Biol..

[195] Li J., Liu Y., Rhee H.S., Ghosh S.K.B., Bai L., Pugh B.F., Gilmour D.S. (2013). Kinetic Competition between Elongation Rate and Binding of NELF Controls Promoter-Proximal Pausing. Mol. Cell.

[196] Cheng B., Price D.H. (2007). Properties of RNA Polymerase II Elongation Complexes Before and After the P-TEFb-Mediated Transition into Productive Elongation. J. Biol. Chem..

[197] Henriques T., Gilchrist D.A., Nechaev S., Bern M., Muse G.W., Burkholder A., Fargo D.C., Adelman K. (2013). Stable Pausing by RNA Polymerase II Provides an Opportunity to Target and Integrate Regulatory Signals. Mol. Cell.

[198] Egloff S. (2021). CDK9 Keeps RNA Polymerase II on Track. Cell. Mol. Life Sci..

[199] Sansó M., Levin R.S., Lipp J.J., Wang V.Y.-F., Greifenberg A.K., Quezada E.M., Ali A., Ghosh A., Larochelle S., Rana T.M. (2016). P-TEFb Regulation of Transcription Termination Factor Xrn2 Revealed by a Chemical Genetic Screen for Cdk9 Substrates. Genes Dev..

[200] Decker T.-M., Forné I., Straub T., Elsaman H., Ma G., Shah N., Imhof A., Eick D. (2019). Analog-Sensitive Cell Line Identifies Cellular Substrates of CDK9. Oncotarget.

[201] Wagner E.J., Tong L., Adelman K. (2023). Integrator Is a Global Promoter-Proximal Termination Complex. Mol. Cell.

[202] Jonkers I., Kwak H., Lis J.T. (2014). Genome-Wide Dynamics of Pol II Elongation and Its Interplay with Promoter Proximal Pausing, Chromatin, and Exons. eLife.

[203] Chao S.-H., Price D.H. (2001). Flavopiridol Inactivates P-TEFb and Blocks Most RNA Polymerase II Transcription in Vivo. J. Biol. Chem..

[204] Chen F.X., Smith E.R., Shilatifard A. (2018). Born to Run: Control of Transcription Elongation by RNA Polymerase II. Nat. Rev. Mol. Cell Biol..

[205] Zhou Q., Li T., Price D.H. (2012). RNA Polymerase II Elongation Control. Annu. Rev. Biochem..

[206] Izban M.G., Luse D.S. (1992). Factor-Stimulated RNA Polymerase II Transcribes at Physiological Elongation Rates on Naked DNA but Very Poorly on Chromatin Templates. J. Biol. Chem..

[207] Conaway R.C., Conaway J.W. (2019). The Hunt for RNA Polymerase II Elongation Factors: A Historical Perspective. Nat. Struct. Mol. Biol..

[208] Luo Z., Lin C., Shilatifard A. (2012). The Super Elongation Complex (SEC) Family in Transcriptional Control. Nat. Rev. Mol. Cell Biol..

[209] Chen Y., Vos S.M., Dienemann C., Ninov M., Urlaub H., Cramer P. (2021). Allosteric Transcription Stimulation by RNA Polymerase II Super Elongation Complex. Mol. Cell.

[210] Van Oss S.B., Cucinotta C.E., Arndt K.M. (2017). Emerging Insights into the Roles of the Paf1 Complex in Gene Regulation. Trends Biochem. Sci..

[211] Francette A.M., Tripplehorn S.A., Arndt K.M. (2021). The Paf1 Complex: A Keystone of Nuclear Regulation Operating at the Interface of Transcription and Chromatin. J. Mol. Biol..

[212] Mbogning J., Nagy S., Pagé V., Schwer B., Shuman S., Fisher R.P., Tanny J.C. (2013). The PAF Complex and Prf1/Rtf1 Delineate Distinct Cdk9-Dependent Pathways Regulating Transcription Elongation in Fission Yeast. PLoS Genet..

[213] Qiu H., Hu C., Gaur N.A., Hinnebusch A.G. (2012). Pol II CTD Kinases Bur1 and Kin28 Promote Spt5 CTR-Independent Recruitment of Paf1 Complex: Dual Pathway of Paf1C Recruitment. EMBO J..

[214] Wier A.D., Mayekar M.K., Héroux A., Arndt K.M., VanDemark A.P. (2013). Structural Basis for Spt5-Mediated Recruitment of the Paf1 Complex to Chromatin. Proc. Natl. Acad. Sci. USA.

[215] Liu Y., Warfield L., Zhang C., Luo J., Allen J., Lang W.H., Ranish J., Shokat K.M., Hahn S. (2009). Phosphorylation of the Transcription Elongation Factor Spt5 by Yeast Bur1 Kinase Stimulates Recruitment of the PAF Complex. Mol. Cell. Biol..

[216] Yu M., Yang W., Ni T., Tang Z., Nakadai T., Zhu J., Roeder R.G. (2015). RNA Polymerase II–Associated Factor 1 Regulates the Release and Phosphorylation of Paused RNA Polymerase II. Science.

[217] Lu X., Zhu X., Li Y., Liu M., Yu B., Wang Y., Rao M., Yang H., Zhou K., Wang Y. (2016). Multiple P-TEFbs Cooperatively Regulate the Release of Promoter-Proximally Paused RNA Polymerase II. Nucleic Acids Res..

[218] Chen F.X., Woodfin A.R., Gardini A., Rickels R.A., Marshall S.A., Smith E.R., Shiekhattar R., Shilatifard A. (2015). PAF1, a Molecular Regulator of Promoter-Proximal Pausing by RNA Polymerase II. Cell.

[219] Chen F.X., Xie P., Collings C.K., Cao K., Aoi Y., Marshall S.A., Rendleman E.J., Ugarenko M., Ozark P.A., Zhang A. (2017). PAF1 Regulation of Promoter-Proximal Pause Release via Enhancer Activation. Science.

[220] Mosimann C., Hausmann G., Basler K. (2006). Parafibromin/Hyrax Activates Wnt/Wg Target Gene Transcription by Direct Association with β-Catenin/Armadillo. Cell.

[221] Kikuchi I., Takahashi-Kanemitsu A., Sakiyama N., Tang C., Tang P.-J., Noda S., Nakao K., Kassai H., Sato T., Aiba A. (2016). Dephosphorylated Parafibromin Is a Transcriptional Coactivator of the Wnt/Hedgehog/Notch Pathways. Nat. Commun..

[222] Skene P.J., Hernandez A.E., Groudine M., Henikoff S. (2014). The Nucleosomal Barrier to Promoter Escape by RNA Polymerase II Is Overcome by the Chromatin Remodeler Chd1. eLife.

[223] Farnung L., Ochmann M., Engeholm M., Cramer P. (2021). Structural Basis of Nucleosome Transcription Mediated by Chd1 and FACT. Nat. Struct. Mol. Biol..

[224] Kulaeva O.I., Hsieh F.-K., Chang H.-W., Luse D.S., Studitsky V.M. (2013). Mechanism of Transcription through a Nucleosome by RNA Polymerase II. Biochim. Biophys. Acta BBA Gene Regul. Mech..

[225] Filipovski M., Soffers J.H.M., Vos S.M., Farnung L. (2022). Structural Basis of Nucleosome Retention during Transcription Elongation. Science.

[226] Squazzo S.L. (2002). The Paf1 Complex Physically and Functionally Associates with Transcription Elongation Factors in Vivo. EMBO J..

[227] Krogan N.J., Kim M., Ahn S.H., Zhong G., Kobor M.S., Cagney G., Emili A., Shilatifard A., Buratowski S., Greenblatt J.F. (2002). RNA Polymerase II Elongation Factors of Saccharomyces Cerevisiae: A Targeted Proteomics Approach. Mol. Cell. Biol..

[228] Lee Y., Park D., Iyer V.R. (2017). The ATP-Dependent Chromatin Remodeler Chd1 Is Recruited by Transcription Elongation Factors and Maintains H3K4me3/H3K36me3 Domains at Actively Transcribed and Spliced Genes. Nucleic Acids Res..

[229] Warner M.H., Roinick K.L., Arndt K.M. (2007). Rtf1 Is a Multifunctional Component of the Paf1 Complex That Regulates Gene Expression by Directing Cotranscriptional Histone Modification. Mol. Cell. Biol..

[230] Laribee R.N., Krogan N.J., Xiao T., Shibata Y., Hughes T.R., Greenblatt J.F., Strahl B.D. (2005). BUR Kinase Selectively Regulates H3 K4 Trimethylation and H2B Ubiquitylation through Recruitment of the PAF Elongation Complex. Curr. Biol..

[231] Xiao T., Kao C.-F., Krogan N.J., Sun Z.-W., Greenblatt J.F., Osley M.A., Strahl B.D. (2005). Histone H2B Ubiquitylation Is Associated with Elongating RNA Polymerase II. Mol. Cell. Biol..

[232] Fetian T., McShane B.M., Horan N.L., Shodja D.N., True J.D., Mosley A.L., Arndt K.M. (2023). Paf1 Complex Subunit Rtf1 Stimulates H2B Ubiquitylation by Interacting with the Highly Conserved N-Terminal Helix of Rad6. Proc. Natl. Acad. Sci. USA.

[233] Heidemann M., Hintermair C., Voß K., Eick D. (2013). Dynamic Phosphorylation Patterns of RNA Polymerase II CTD during Transcription. Biochim. Biophys. Acta BBA Gene Regul. Mech..

[234] Yoh S.M., Cho H., Pickle L., Evans R.M., Jones K.A. (2007). The Spt6 SH2 Domain Binds Ser2-P RNAPII to Direct Iws1-Dependent mRNA Splicing and Export. Genes Dev..

[235] David C.J., Boyne A.R., Millhouse S.R., Manley J.L. (2011). The RNA Polymerase II C-Terminal Domain Promotes Splicing Activation through Recruitment of a U2AF65–Prp19 Complex. Genes Dev..

[236] Morris D.P., Greenleaf A.L. (2000). The Splicing Factor, Prp40, Binds the Phosphorylated Carboxyl-Terminal Domain of RNA Polymerase II. J. Biol. Chem..

[237] MacKellar A.L., Greenleaf A.L. (2011). Cotranscriptional Association of mRNA Export Factor Yra1 with C-Terminal Domain of RNA Polymerase II. J. Biol. Chem..

[238] Mayer A., Heidemann M., Lidschreiber M., Schreieck A., Sun M., Hintermair C., Kremmer E., Eick D., Cramer P. (2012). CTD Tyrosine Phosphorylation Impairs Termination Factor Recruitment to RNA Polymerase II. Science.

[239] Schreieck A., Easter A.D., Etzold S., Wiederhold K., Lidschreiber M., Cramer P., Passmore L.A. (2014). RNA Polymerase II Termination Involves C-Terminal-Domain Tyrosine Dephosphorylation by CPF Subunit Glc7. Nat. Struct. Mol. Biol..

[240] Shah N., Maqbool M.A., Yahia Y., El Aabidine A.Z., Esnault C., Forné I., Decker T.-M., Martin D., Schüller R., Krebs S. (2018). Tyrosine-1 of RNA Polymerase II CTD Controls Global Termination of Gene Transcription in Mammals. Mol. Cell.

[241] Hintermair C., Heidemann M., Koch F., Descostes N., Gut M., Gut I., Fenouil R., Ferrier P., Flatley A., Kremmer E. (2012). Threonine-4 of Mammalian RNA Polymerase II CTD Is Targeted by Polo-like Kinase 3 and Required for Transcriptional Elongation: CTD Thr4 Is Required for Transcription Elongation. EMBO J..

[242] Rodríguez-Molina J.B., West S., Passmore L.A. (2023). Knowing When to Stop: Transcription Termination on Protein-Coding Genes by Eukaryotic RNAPII. Mol. Cell.

[243] Xie J., Libri D., Porrua O. (2023). Mechanisms of Eukaryotic Transcription Termination at a Glance. J. Cell Sci..

[244] Eaton J.D., West S. (2020). Termination of Transcription by RNA Polymerase II: BOOM!. Trends Genet..

[245] Proudfoot N.J. (2016). Transcriptional Termination in Mammals: Stopping the RNA Polymerase II Juggernaut. Science.

[246] Cortazar M.A., Sheridan R.M., Erickson B., Fong N., Glover-Cutter K., Brannan K., Bentley D.L. (2019). Control of RNA Pol II Speed by PNUTS-PP1 and Spt5 Dephosphorylation Facilitates Termination by a “Sitting Duck Torpedo” Mechanism. Mol. Cell.

[247] Wu X., Brewer G. (2012). The Regulation of mRNA Stability in Mammalian Cells: 2.0. Gene.

[248] Neve J., Patel R., Wang Z., Louey A., Furger A.M. (2017). Cleavage and Polyadenylation: Ending the Message Expands Gene Regulation. RNA Biol..

[249] Nordick K., Hoffman M.G., Betz J.L., Jaehning J.A. (2008). Direct Interactions between the Paf1 Complex and a Cleavage and Polyadenylation Factor Are Revealed by Dissociation of Paf1 from RNA Polymerase II. Eukaryot. Cell.

[250] Penheiter K.L., Washburn T.M., Porter S.E., Hoffman M.G., Jaehning J.A. (2005). A Posttranscriptional Role for the Yeast Paf1-RNA Polymerase II Complex Is Revealed by Identification of Primary Targets. Mol. Cell.

[251] Nagaike T., Logan C., Hotta I., Rozenblatt-Rosen O., Meyerson M., Manley J.L. (2011). Transcriptional Activators Enhance Polyadenylation of mRNA Precursors. Mol. Cell.

[252] Katahira J. (2015). Nuclear Export of Messenger RNA. Genes.

[253] Logan J., Falck-Pedersen E., Darnell J.E., Shenk T. (1987). A Poly(A) Addition Site and a Downstream Termination Region Are Required for Efficient Cessation of Transcription by RNA Polymerase II in the Mouse Beta Maj-Globin Gene. Proc. Natl. Acad. Sci. USA.

[254] Zhang H., Rigo F., Martinson H.G. (2015). Poly(A) Signal-Dependent Transcription Termination Occurs through a Conformational Change Mechanism That Does Not Require Cleavage at the Poly(A) Site. Mol. Cell.

[255] Eaton J.D., Francis L., Davidson L., West S. (2020). A Unified Allosteric/Torpedo Mechanism for Transcriptional Termination on Human Protein-Coding Genes. Genes Dev..

